# A c-Myc-regulated stem cell-like signature in high-risk neuroblastoma: A systematic discovery (Target neuroblastoma ESC-like signature)

**DOI:** 10.1038/s41598-017-00122-x

**Published:** 2017-03-03

**Authors:** Xinan (Holly) Yang, Fangming Tang, Jisu Shin, John M. Cunningham

**Affiliations:** Section of Hematology and Oncology, Department of Pediatrics, University of Chicago, Chicago, IL 60637 USA

## Abstract

c-Myc dysregulation is hypothesized to account for the ‘stemness’ – self-renewal and pluripotency – shared between embryonic stem cells (ESCs) and adult aggressive tumours. High-risk neuroblastoma (HR-NB) is the most frequent, aggressive, extracranial solid tumour in childhood. Using HR-NB as a platform, we performed a network analysis of transcriptome data and presented a c-Myc subnetwork enriched for genes previously reported as ESC-like cancer signatures. A subsequent drug-gene interaction analysis identified a pharmacogenomic agent that preferentially interacted with this HR-NB-specific, ESC-like signature. This agent, Roniciclib (BAY 1000394), inhibited neuroblastoma cell growth and induced apoptosis *in vitro*. It also repressed the expression of the oncogene c-Myc and the neural ESC marker CDK2 *in vitro*, which was accompanied by altered expression of the c-Myc-targeted cell cycle regulators *CCND1, CDKN1A* and *CDKN2D* in a time-dependent manner. Further investigation into this HR-NB-specific ESC-like signature in 295 and 243 independent patients revealed and validated the general prognostic index of CDK2 and CDKN3 compared with CDKN2D and CDKN1B. These findings highlight the very potent therapeutic benefits of Roniciclib in HR-NB through the targeting of c-Myc-regulated, ESC-like tumorigenesis. This work provides a hypothesis-driven systems computational model that facilitates the translation of genomic and transcriptomic signatures to molecular mechanisms underlying high-risk tumours.

## Introduction

Evidence from heterogeneous primary tumours suggests the common presence of a rare cellular subpopulation, termed tumour-initiating cells, that exhibits self-renewal and chemo-resistance properties reminiscent of those of normal stem cells^1–4^. The phenotypes of a specific type of normal stem cell, embryonic stem cells (**ESCs**), have frequently been associated with the molecular and functional characteristics of several aggressive and treatment resistant tumours^5, 6^. Characterizing the transcriptome of ESCs is critical for understanding treatment-resistance because RNAs -﻿ ﻿wh﻿ich ﻿are transcribed from the genome and traslated into the p﻿r﻿otein - ﻿play a key mediating role between different cellular networks (here the genome and the proteome). As such, how systematic computational approaches can help depict the **ESC-like, cancer-activated** gene signatures that underpin the translational regulatory network shared by heterogeneous aggressive tumours remains unclear.

Neuroblastoma (**NB**) is the most common extracranial solid tumour in children. Patients with high-risk (**HR**) NB exhibit greater than 60% mortality and recurrence rates^7^. This tumour cell population is resistant to chemo- and radiotherapy and often possesses the functional and molecular characteristics of normal stem cells. Therefore, this study used HR-NB as a model to systematically investigate ESC-like oncogenic mechanisms, although our approach is applicable to other malignant tumours.

All NB patients with an amplification of the *MYCN* oncogene (**MA**) are considered high-risk^8, 9^. However, many HR-NB tumours harbour a normal MYCN locus, termed MYCN-non-amplified (**MN**) NB, suggesting the existence of other genetic markers. Approximately 20% and 11% of HR-NB cases show augmented expression of the MYC family members MYCN and c-Myc, respectively, both of which correlate with poor prognosis^10–12^. Particularly, c-Myc is well known as a master regulator of stemness and the pathophysiology of cancer^5, 6, 13^. c-Myc-interacting proteins are responsible for most of the similarities between ESCs and several aggressive tumours, although this finding has yet to be shown in HR-NB^14^. Therefore, deciphering the role of c-Myc in this high-risk state as well as the associated functional and molecular characteristics of ESCs in HR-NB, will offer a comprehensive understanding of malignant translational regulation.

We hypothesized that systems approaches could aggregate the flow of biological information and discover a common mechanism underlying outcomes in HR-NB, given that diverse genetic and transcriptomic disturbances can lead to malignancies with similar phenotypes. Cancer cells have deviated from the normal genome by acquiring and selecting for a set of mutations that enable their malignancy^15^. These changes can be germline variations, somatic mutations, or upstream deregulations that trigger carcinogenesis. However, evaluating individual genomic features often does not reveal significant signatures. Thus, systems biology has emerged as a promising direction to translate cancer genomics into precision medicine by integrating experimental and computational research^16–20^. One systems-bioinformatics approach, namely, protein-protein interaction (**PPI**) analysis, has successfully pinpointed and interpreted functional mutations or dysregulations in several diseases, including HR-NB^21–23^. Here, we focus on two topological features of PPIs, termed hub and bottleneck. “**Hub**” proteins are highly connected in complex networks and tend to be more essential than non-hub proteins^21^, whereas “**bottleneck**” proteins retain the shortest paths (analogous to major bridges and tunnels on a highway map) and correlate with gene essentiality and expression dynamics^24, 25^.

**Figure 1:**
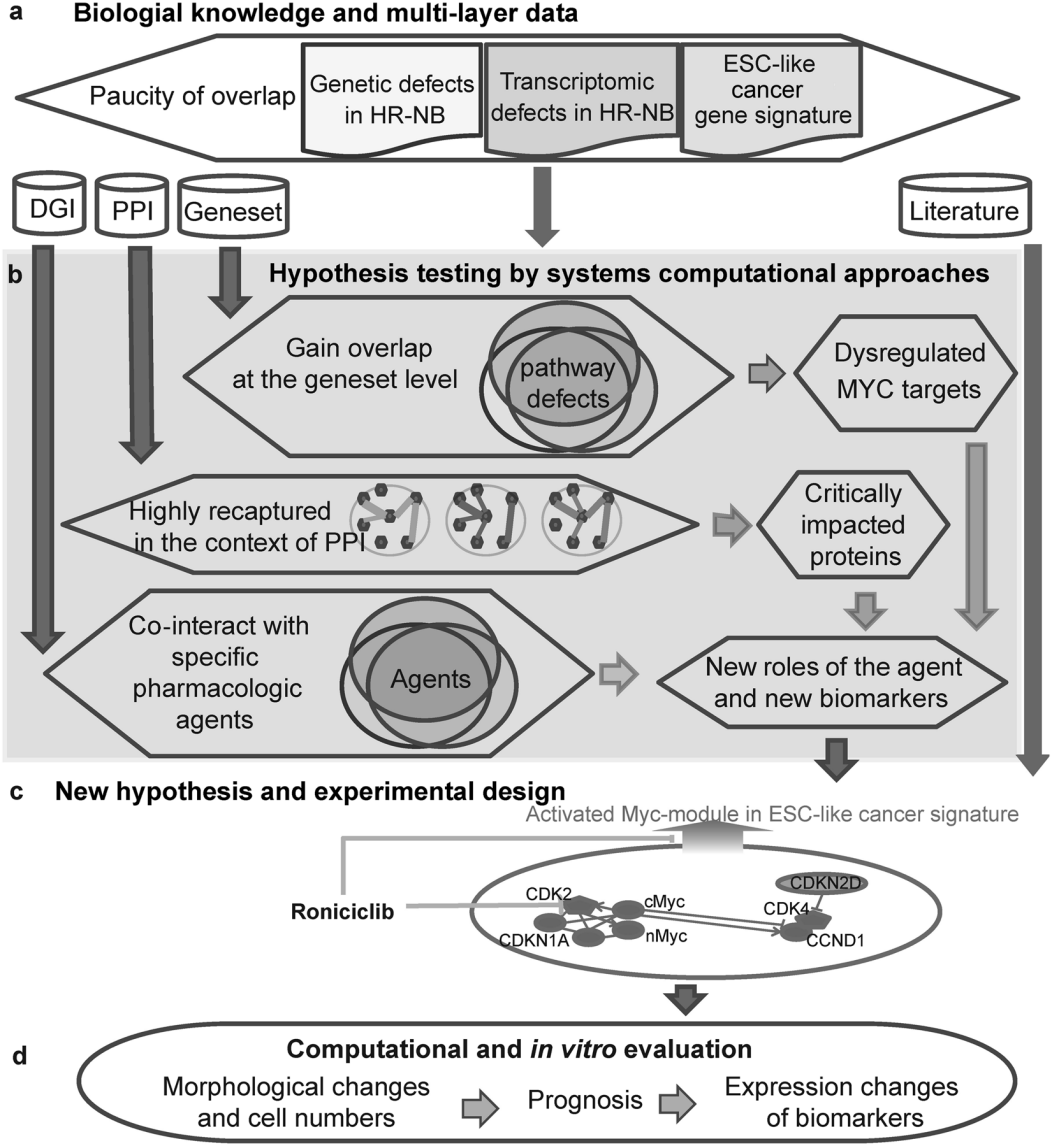
Systems biology analysis workflow. (**a**) Multi-scale data collection (inputs). (**b**) Systems computational approaches for hypothesis testing. Enriched gene-sets gain overlap at the biological pathway level. Thus, we identified recurrently dysregulated pathways and pathway genes from multi-scale data. Subsequently, we identified critical topological features from the HR-NB-associated PPI subnetwork. Finally, we scanned pharmacological agents for the over-representation of their interacting targets among the gene signatures that were recurrent at the pathway and protein levels. Notably, this computational work links clinical relevance to an ESC-like signal. (**c**) Generation of new hypotheses based on the above computational analysis and literature review and the subsequent design of biological experiments. (**d**) Biological *in vitro* evaluation and clinical application validated for data from independent patients.

In this study, we designed a top-down computational systems approach that encompasses regulatory network reconstructions from ‘omics’ data and prior knowledge databases^26^, and validated a predicted pharmacological agent and biomarkers *in vitro* (Fig. 1). We showed common downstream effects of ESC-like signatures that are shared among heterogeneous HR-NB tumours that are frequently dysregulated by MYC proteins in the same signalling pathway. Extending previous work on adult tumours^5^, we identified a c-Myc-regulatory network as the potential common thread connecting ESC-like cancer-activated features to childhood HR-NB. Remarkably, these multi-scale incorporated signatures were able to reveal critical drug targets and identify new applications for existing pharmacologic agents.

## Results

### Altered expression of genes mediated by MYCN is associated with outcomes in HR-NB

We first tried to understand transcriptomic disruption in NB patients from published transcriptomic profiles. To increase the statistical sensitivity, we performed a meta-analysis on the 1,368 collected transcriptomic profiles (Supplementary Table S1)^27^. Among HR patients, we identified 2,321 and 2,104 genes that were up-regulated in MA patients and MN patients, respectively (Stouffer FDR < 0.001). Consistent with previous work^28^, c-Myc was highly expressed in MN patients compared with MA patients (2.3- and 3.5-fold changes measured by Agilent and Affymetrix exon arrays, respectively). Among MN patients, we defined 2,858 “**HR genes**” that were up-regulated in HR patients compared with low-risk (**LR**) patients. These HR genes exhibited significant differences in expression in at least one studied cohort (FDR < 0.05, fold-change >1.3, 1.5 or 1.8). Similarly, we identified 2040 “**LR genes**” that served as controls.

We observed pairwise enrichment among the HR, MA and MN genes and a well-known prognostic 42-gene classifier^29^ (Supplementary Fig. S1A). Specifically, the prognostic 42-gene classifier recaptured both MA and MN genes but consisted of more MN genes (n = 28) than MA genes (n = 14). Notably, the HR genes derived from MN patients covered both the MA and MN genes and significantly overlapped with the MA genes. This overlap suggests the existence of common underlying mechanisms for HR-NB tumours that occur irrespective of MYCN copy number.

We then evaluated the hypothesis that both MA and MN genes have potential clinical applications. Three additional well-known prognostic gene signatures for HR-NB were collected (Supplementary Methods)^29–32^. Supplementary Figure S1 (panels B-C) shows that joint MA and MN genes significantly recaptured prognostic signatures (24, 42 and 105 overlaps, P = 0.0004, <2e-16, <2e-16 for the 52, 42 and 157 gene signatures, respectively; Fisher’s exact test (**FET**)). We concluded that changes in the expression of genes mediated by MYCN, rather than the status of the MYCN locus, are associated with outcomes in HR-NB.

### A subset of n-Myc targets are highly expressed in MN tumours and regulated by c-Myc

We expected that n-Myc interacted with c-Myc and other members of the HR-NB signature, based on the two expression patterns that we identified: first, that c-Myc was over-expressed in MN tumours; and second, that the altered expression of genes mediated by n-Myc was associated with outcomes in all HR-NB cases. To validate this hypothesis, we retrieved 1,259 n-Myc-interacting genes and 1,275 c-Myc-targeted genes from the MSigDB database (Supplementary Table S2). These two sets of MYC targets overlapped significantly (361 co-targeted genes, OR = 8, P < 2e-16). Importantly, both MYC targets were preferentially over-expressed in HR tumours (OR > 1.2; P < 0.02, Supplementary Fig. S2A), suggesting shared MYC oncogenicity.

To understand how transcriptomic and genetic disturbances cooperate to affect MYC oncogenicity, we constructed a subset of protein-protein interactions (**PPIs**). This PPI subnetwork consisted of the MYC targets with verified or recurrent somatic mutations in HR-NB (Supplementary Fig. S2B, the STRING database^33^). We observed that a subset of n-Myc targets was also highly expressed in MN tumours and regulated by the myc homologue c-Myc. Moreover, the oncogene NRAS was among the n-Myc/c-Myc co-targets and interacted with other HR-NB-causing mutations, such as *ALK, TP53* and *BARD1*. Notably, RAS mutation or activation has been reported to enhance MYC oncogenicity (reviewed by^34^). Taken together, understanding the functional roles of c-Myc and n-Myc is equally important for HR-NB because increase in the expression levels of these two proteins independently indicates poor prognosis with similar significance^10–12^.

### c-Myc is a critical hub in the HR-associated subnetwork and interacts with known anti-NB drug targets

To explore frequently dysregulated mechanisms in both MA and MN high-risk tumour, we defined an “**NB-associated subnetwork**” (**Methods**). In this NB-associated PPI subnetwork, 79 protein “hubs” met the significance level in terms of network degree in both the theoretical test (FET P < 1e-5, OR > 2) and an empirical test (P < 0.001). These network “hubs” make the linkage robust against accidental failures but vulnerable to coordinated attacks and thus represent essential regulators in HR-NB.

**Figure 2:**
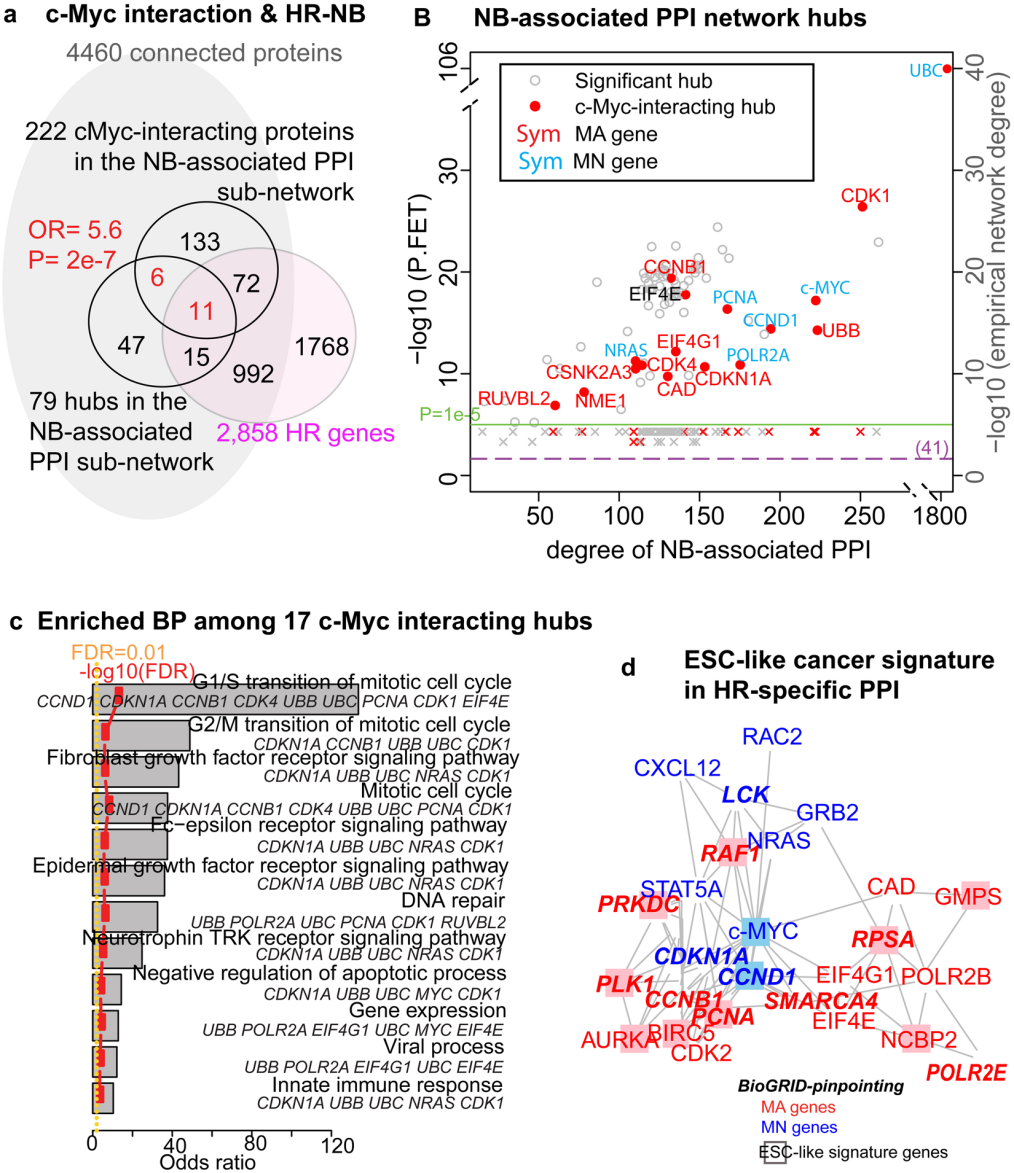
An HR-NB-associated PPI subnetwork suggests that MYC regulation is the common thread linking the cancer-activated ESC-like signature to HR-NB. (**a**) A Venn diagram of c-Myc interacting proteins, hubs in the NB-associated PPI subnetwork, and the HR signature. c-Myc gains significant interactions with other network hubs (P = 2e-7, OR = 5.6), as evidenced by 17 of its 221 neighbours that are hubs (of the 4,460 connected proteins). (**B**) c-Myc is among the leading PPI hubs, with a high network degree that exhibits significance in both the theoretical test (FET P < 1e-5, OR > 2, plotted in nodes) and an empirical test (P < 0.01, plotted in crosses). The green horizontal line indicates the criteria of “hub” (P = 1e-5). The orange dotted horizontal line marks an average degree of 41. The gene symbol colour corresponds to MYCN-dependent over-expression (red: MA, blue: MN; black: NS). (**c**) Seventeen c-Myc-interacting PPI hubs over-represented GO terms for biological-process (BP). The reported biological processes met the following significance criteria: (1) FET odds ratio >2; (2) FET FDR <0.01; (3) FAIME score >0; and 4) at least five gene members. (**d**) The HR-specific PPI sub-network identified 26 proteins as both hubs and bottlenecks (STRING), eleven of which are featured as hubs and bottlenecks using BioGRID (the bold italic symbols). Eleven of these proteins are part of the ESC-like cancer signature (the boxed nodes). Gene symbols are coloured as in panel B.

Among these hubs, c-Myc was directly connected to 221 other proteins in the NB-associated PPI network, irrespective of their MA-dependent dysregulation (Fig. 2a). In contrast, the average degree of 4,460 randomly sampled proteins (out of 15,554 proteins in the entire PPI network (score > 0.7)) was only 41. Additionally, c-Myc frequently interacted with other network hubs (17 of its 221 neighbours were hubs, P = 2e-7 OR = 5.6). These 17 c-Myc-interacting, NB-associated PPI hubs were preferentially overexpressed in HR tumours (11 HR genes, OR = 70, P = 5.6e-14), suggesting an oncogenic role for c-Myc in HR-NB.

We also examined whether c-Myc prefers MA-dependent targets (Fig. 2b), and found that the 17 c-Myc-interacting hubs showed no preference for MYCN-amplification. Particularly, of the 11 c-Myc-interacting HR over-expressed PPI hubs, *CCND1, CDKN1A, MYC,* and *NRAS*, are MN gene products. Notably, most of these 11 genes have been studied as anti-NB drug targets, thus highlighting the critical role of c-Myc in HR-NB (Table 1).

**Table 1:** Most of the 11 c-Myc-interacting, HR signature genes have been studied as anti-NB drug targets, further highlighting the critical role of c-Myc in HR-NB.

Gene Symbol	MYCN-dep.	Drug	Pubmed	Experimental cells	Response to drug(s)	Potential mechanism
**CAD**	MA > MN	N-L-aspartate (PALA)	9418900	REF52 cells	amplification	increase expression of n-Myc and overcome the PALA-induced cell cycle block
**CCNB1**	MA > MN	Lupiwighteone; Thymoquinone	25661352; 26745078	SY5Y; Neuro-2a	decrease/decrease	induce G2/M phase arrest
**EIF4E**	MA > MN	MNK inhibition	25743081	lung etc	inhibition	MNK inhibitors inhibit eIF4E phosphorylation, colony formation, epithelial-to-mesenchymal transition, invasion and MCL1 expression in cancer
**EIF4G1**	MA > MN	NBT-272	20889731	SH-SY5Y xenograft model	interacting partner EIF4E is inhibited	arrest tumour growth through AKT and the MEK/extracellular signal-regulated kinase pathways
**PCNA**	MA > MN	Thymoquinone	26745078	Neuro-2a	decrease	arrest the cell-cycle in the G2/M phase and induce apoptosis
**RUVBL2**	MA > MN	PCI-24781	23977108	SK-N-DZ	knockdown of RuvBL2 rescued cells from PCI-24781-induced cell death	histone deacetylase inhibitor
**CCND1**	MN > MA	Lupiwighteone	25661352	SY5Y	decrease	induce G2/M phase arrest
**CDKN1A**	MN > MA	Doxorubicin	26284262	IMR-32	decrease	blocking an enzyme called topo isomerase 2 that cancer cells need to divide and grow
**MYC**	MN > MA	PD166285 and 3-DAZA	25477524	SK-N-AS, SH-SY5Y, IMR-32, SK-NBE (2), LAN1, NB19	decrease the expression of AHCY, BLM, PKMYT1, and CKS1B	growth arrest in the G1 phase of the cell cycle of MYCN-negative
**NRAS**	MN > MA	trametinib, GSK2126458, GSK690693	25277205	SK-N-AS, CHP212 and SK-N-DZ	Decrease the NRAS expression	MEK and PI3K/mTOR inhibitor synergistically decreases cell viability in NRAS Q61 mutant NB cell lines

Finally, we analysed the function of these 17 c-Myc-interacting PPI hubs (Fig. 2c). A biological-processes enrichment analysis pinpointed cyclin-dependent kinase (**CDK**) 1, which processes the G2/M transition of the mitotic cell cycle (GO:0000086), and CDK4, which processes the G1/S transition in mitosis (GO:0000082). Other enriched results included neurotrophin-possessed tyrosine kinase activity, which orchestrates sympathetic nervous system development. A negative regulator of apoptosis (GO:0043066) was also enriched among these c-Myc-modulated hubs (CDK1, CDKN1A, MYC, UBB, and UBC). We concluded that c-Myc is critical for mediating the NB-associated PPI regulatory network and impacts both the cell cycle and cell apoptosis.

### A c-Myc interactome characterized by ESC-like cancer-activated signatures

Two results led us to hypothesize that c-Myc is a core component of the ESC-like signature in HR-NB. First, we have shown that a subset of n-Myc targets is also highly expressed in MN tumours and is regulated by c-Myc. Second, we also showed that c-Myc acts as a critical hub in the HR-NB-specific PPI network. These findings highlight the malignancy and treatment resistance shared by MA and MN tumours. To test this hypothesis computationally, we first defined an “**ESC-like cancer signature**” of 646 genes that were merged from two published studies^5, 6^ (Supplementary Fig. S3A). The MYC module in one study significantly over-represents the cancer-activated ESC-like module (P < 2e-16, OR = 10), but not the core ESC nor the PRC module in the other study.

In the HR-NB-associated PPI subnetwork, c-Myc interacted with the regulatory component of the ESC-like cancer signature more frequently than expected (41 interactors, P = 1.3e-7, OR = 2.9, Supplementary Fig. S3B). Additionally, c-Myc and n-Myc significantly targeted the ESC-like cancer signature (FET P < 2e-16, OR = 2.9 and 5, respectively, Supplementary Fig. S3C﻿). These observations suggest a c-Myc subnetwork in which a common ESC-like signature underpins the high-risk state in NB, which results in transcriptomic features that endow tumour-initiating cells.

Next, we examined the ability of an “HR-NB-specific” PPI subnetwork to pinpoint known hallmarks in drug-resistant NB cells (Fig. 2d, Methods). This HR-NB-specific PPI subnetwork identified 26 proteins as both hubs and bottlenecks, including known hallmarks of drug-resistant NB cells that exhibit the self-renewal characteristic of tumour initiating-cells such as, c-Myc^35–39^. This result confirmed that some PPI hubs can act as bottlenecks and significantly affect expression dynamics^25^. Moreover, 17 cell cycle markers, including CDK2, CCND1 and CDKN1A, 20 cellular growth and proliferation markers, and other biomarkers for NB or other tumours, were also identified. We further successfully verified half of these identifications using the same method based on the interactome database BioGRID^40^, which also curates genetic interactions (Supplementary Table S3).

### Predictive modelling suggests that the CDK inhibitor Roniciclib may serve as a potential component of HR-NB therapy

**Figure 3:**
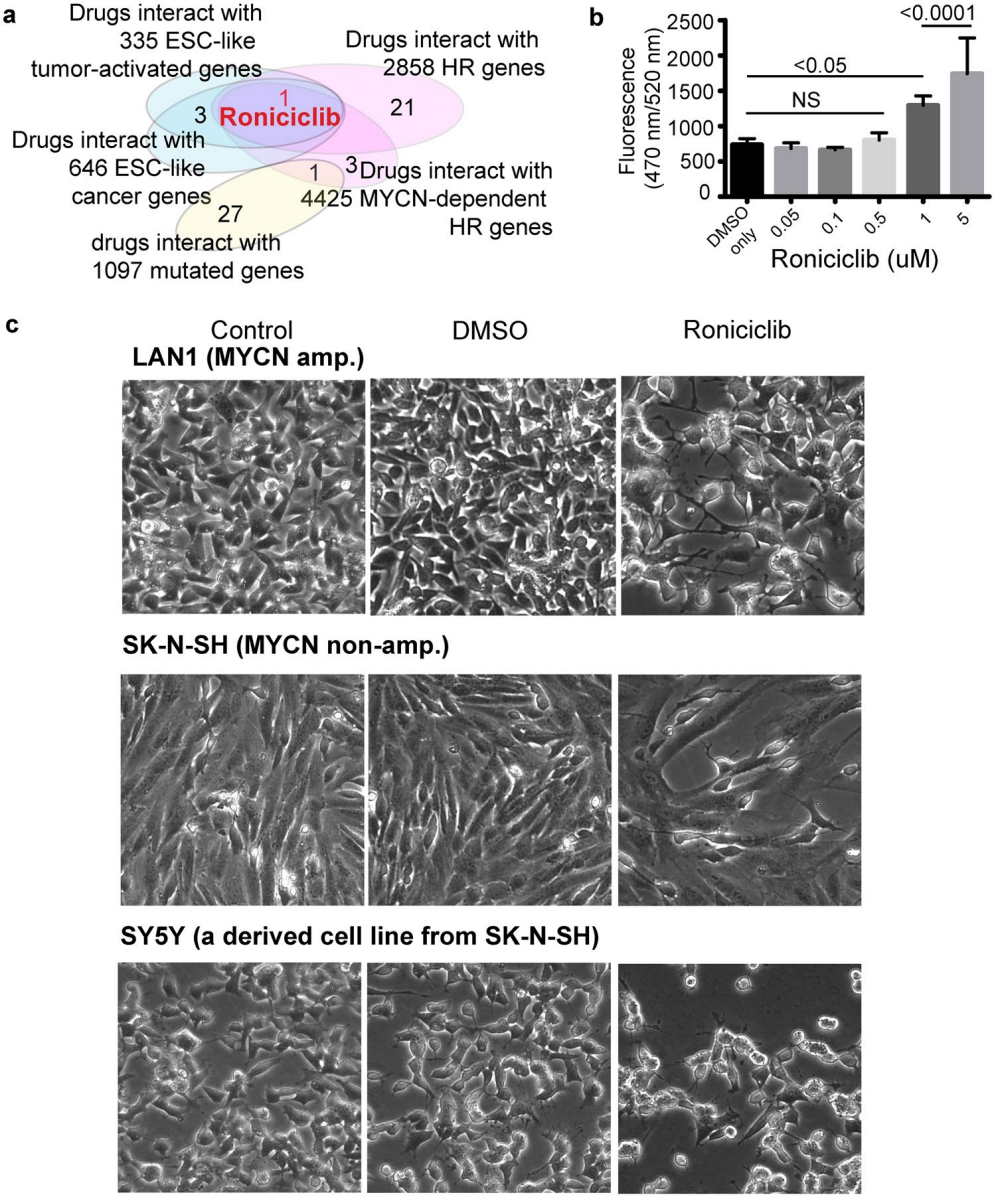
The cyclin-dependent kinase inhibitor Roniciclib induces morphological changes and cell apoptosis in human neuroblastoma cells. (**a**) Roniciclib is the only drug that is commonly over-represented in the ESC-like cancer-activated signature, HR genes, and the MA-dependently expressed MA or MN genes. (**b**) Roniciclib induces apoptotic cell death in SY5Y in a concentration-dependent manner. Proliferating SY5Y cells were exposed to different concentrations of Roniciclib for 72 h. Caspase-3 activity was measured by monitoring Ac-DEVD-R110 fluorescence. The data are represented as the mean ± SD of three independent experiments. (**c**) Morphological changes and cell number decrease in human neuroblastoma cells observed under an inverted microscope (magnification, 20﻿x): Blank control group, dimethyl sulfoxide-treated group (0.1% DMSO, 72 h), and Roniciclib-treated group (1 μmol/L, 72 h).

These computational analyses all showed that c-Myc is associated with an ESC-like, cancer-activated signature in heterogeneous HR-NB tumours in the NB-associated PPI. We also demonstrated that the known hallmarks of drug-resistant NB cells intersected with the HR-NB-specific PPI subnetwork together with c-Myc, irrespective of the MYCN status. These results led us to investigate the biological relevance of pharmacological agents that inhibit key HR-NB oncogenic signatures (Fig. 3a).

By examining the 552 drugs in the drug-gene interaction database (**DGIdb**)^41^, we found that the drug Roniciclib significantly interacted with all three gene signatures: the ESC-like cancer genes, the MYCN-dependently expressed genes and the HR genes (FDR < 0.05, OR > 2, with five or more intersection of genes). **Roniciclib (BAY 1000394)** is a novel oral cytotoxic agent against cell-cycle and transcriptional cyclin-dependent kinases (**CDKs**)^42,43,48^. Roniciclib is currently in Phase II clinical development as a first-line therapy in combination with chemotherapy for extensive small cell lung cancer. To the best of our knowledge, we are the first to report the impact of Roniciclib on HR-NB.

To determine the effects of Roniciclib treatment on cellular apoptosis, we measured Caspase-3-like activity in the SY5Y cells pre- and post-therapy (Supplementary Methods). Treatment with 1 μmol/L Roniciclib for 72 h significantly reduced the number of cells compared with the DMSO-treated control, as analysed by fluorometric HTS assay Kit (P < 0.05, Fig. 3b, three replicates). Consistent with these results, standard cell proliferation assays showed that the relative proliferation of cells decreased to approximately 50% in response to Roniciclib (1 μmol/L, 72 h, three replicates) (Supplementary Fig. S4 and Supplementary Methods).

To investigate the general impact of Roniciclib on NB tumour apoptosis, we compared the cell proliferation and morphology of Roniciclib-treated cells with vehicle or control-treated MA (LAN1) and MN (SY5Y and SK-N-SH) cells (Fig. 3c). Roniciclib treatment resulted in morphological changes in all three NB cell lines and inhibited NB cell growth at 1 μM. Microscopy revealed fewer cells and major morphological changes after Roniciclib treatment. After 72 h of incubation, the number of cells was significantly reduced in Roniciclib-treated wells compared with vehicle treatment. Additionally, we observed apoptotic characteristics, including cell shrinkage and detachment from the surrounding tissue. We concluded that exposure to Roniciclib induces differentiation (as seen by changes in morphology), a reduction in cell proliferation, and increased apoptosis in HR-NB cell lines. These interesting observations suggest that this molecule may have efficacy and should be tested in multimodal HR-NB therapy.

### A subset of CDK- and CDKN-family genes potentially marks ESC-like NB-initiating mechanisms and thus indicates poor outcomes for HR-NB, and these genes were highly activated in both HR-NB and embryonic neural stem cells

**Figure 4:**
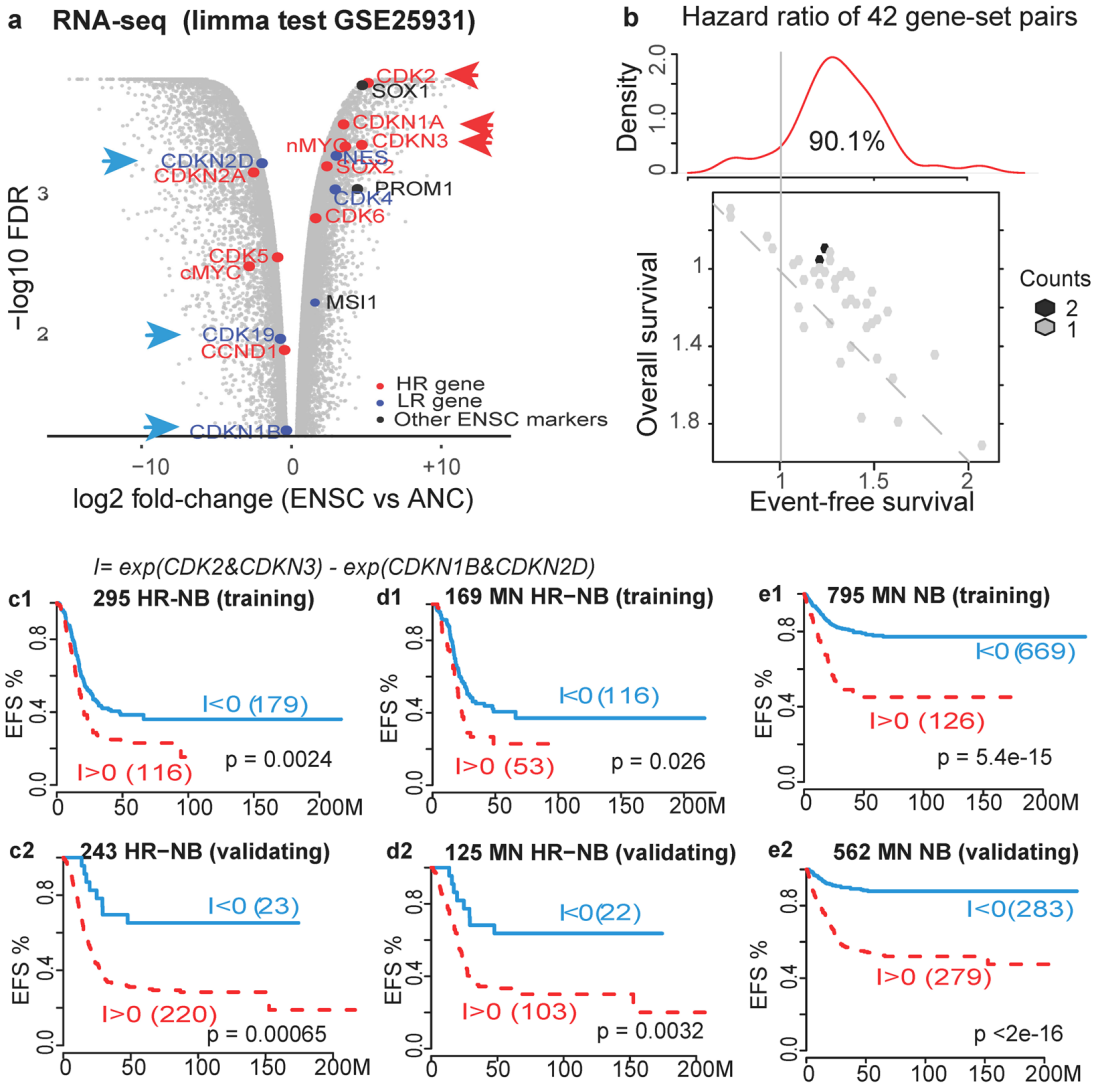
Identification of prognostic biomarkers and clinical evaluation. (**a**) The identification of prognostic CDK and CDKN genes (indicated by arrows). The genes indicated in red are poor-outcome candidates among HR-signature genes that are highly expressed in embryonic neural stem cells (ENSCs) compared with adult neural cell (ANC) samples (log2 FC > 1.5). The genes indicated in blue are good-outcome candidates among the LR genes that are highly expressed in ANC compared with ENSC. (**b**) Hazard ratios of survival analysis for all 42 possible gene-set pairs. The top subpanel shows the density of the hazard ratio distribution for the event-free survival (EFS) analysis. A value larger than 1 indicates an unfavourable outcome. The bottom subpanel is a hex-binned heatmap of EFS hazard ratios compared with overall survival (OS) hazard ratios. The dashed line indicates two analyses that have the same hazard ratios. (**c**,**d﻿,e﻿)** Kaplan-Meier plots for the EFS of patients. The subpanels c-e depict patients with HR-NB, HR-NB patients without MYCN amplification (MN), and all NB patients without MYCN amplification, respectively. Subpanels 1 and 2 depict the training and validation datasets, respectively. In all subpanels, positive I-scores (i.e., higher CDK2 and CDKN3 expression compared with CDKN1B and CDKN2D) significantly indicate poor outcome for HR-NB (P < 0.005).

Translating transcriptomic ESC-like signatures into clinical applications in HR-NB is highly important. To identify a tumour initiating cell-like signature in HR-NB, we overlaid the normal embryonic neural stem cell (**ENSC**) signature with the HR genes (Fig. 4a). Furthermore, we compared CDKs and CDKNs that have been identified as either HR or LR genes because the CDK-inhibitor Roniciclib significantly targets ESC-like signature genes in HR-NB. By re-analyzing transcriptomic profiles of human ENSCs and a multicellular adult tissue rich in neurons (GSE25931)^44^, we successfully verified the high expression of ENSC markers. Moreover, the HR genes *CDK2, CDKN1A* and *CDKN3* were among the leading highly expressed ENSC genes, suggesting that they represent ‘stemness’ properties in HR-NB cells. Furthermore, the LR genes *CDKN2D, CDK19* and *CDKN1B* were among the ENSC down-regulated genes, suggesting that they represent the differentiated status of NB cells. Consistent with these observations, CDKN1B and CDKN2D have been reported as NB tumour suppressors^45, 46^. Therefore, we expected the relative expression levels of these two sets of biomarkers to indicate an unfavourable outcome in HR-NB.

To evaluate the prognostic effects of the above newly identified biomarkers, we applied our previously proposed approach that addresses the challenge of using biomarker expression across multiple platforms and making the values comparable^47^. This algorithm, relative expression analysis of gene-set pair (**RXA-GSP**), quantifies the systematic imbalance between poor-outcome components and good-outcome components in patients, and this individualized prognostic index is applicable to multiple platforms. In this study, all tested indexes resulted in predominately positive hazard ratios, indicating poor event-free survival (EFS) and confirming the identification of individualized poor-outcome markers (Fig. 4b). Additionally, the EFS analysis resulted in a higher hazard ratio than the over-all survival analysis, suggesting a potential association between these GSPs and treatment resistance.

We then identified seven gene-set pairs whose indexes (I-score) significantly predicted unfavourable event-free outcomes in the training cohort (P < 0.005, hazard ratio >1.5). Additionally, we successfully validated the prognostic power of these seven indexes using the expression profiles of 243 patients with newly diagnosed HR-NB^48, 49^ (P < 0.05, hazard ratio >1.5, Table 2). In contrast, MYCN-amplification only moderately stratified the event-free survival in these two cohorts (P = 0.008 and 0.025, Supplementary Fig. S5) and did not stratify MN patients.

**Table 2:** Seven prognostic gene-set pairs (GSPs) consisting of poor-outcome markers (CDK2, CDKN1A and CDKN3) and good-outcome markers (CDKN2D, CDK19 and CDKN1B).

Poor-outcome	Good-outcome	Training dataset				Validation dataset			
		# of pts with I < 0	# of pts with I > 0	EFS P-value	ESP hazard ratio	# of pts with I < 0	# of pts with I > 0	EFS P-value	ESP hazard ratio
CDKN3	CDKN2D	136	169	1.5E-06	2.07	30	219	0.017	1.89
CDKN3	CDK19, CDKN2D	162	143	3.4E-05	1.83	21	228	0.0021	3.10
CDKN3	CDKN1B, CDKN2D	183	122	0.0044	1.52	23	226	0.0131	2.21
CDK2, CDKN3	CDKN2D	143	162	0.0046	1.52	44	205	0.007	1.82
CDKN1A, CDKN3	CDKN2D	138	167	0.0016	1.60	107	142	0.0058	1.56
CDK2, CDKN3	CDKN1B, CDKN2D	189	116	0.0024	1.57	24	225	0.0007	3.24
CDK2, CDKN1A, CDKN3	CDKN2D	136	169	0.0013	1.61	83	166	0.0044	1.62

The strongest prognostic power (P = 0.0024 and 0.00065, Fig. 4c) came from the gene-set pair of two ENSC markers (CDK2 and CDKN3^44^) with two NB tumour suppressors (*CDKN1B* and *CDKN2D* ^45, 46^). Moreover, this gene-set pair stratified event-free survival for patients with normal MYCN copy numbers (MN) in the HR populations (P = 0.026, 0.0032, Fig. 4d) and the overall MN populations (P = 5e-15 and <2e-16, Fig. 4e). We concluded that this prognostic index of CDK and CDKN family genes is independent of and more powerful than the MYCN amplification status.

### Roniciclib inhibits c-Myc and CDK2 while increasing CCND1 in HR-NB

**Figure 5:**
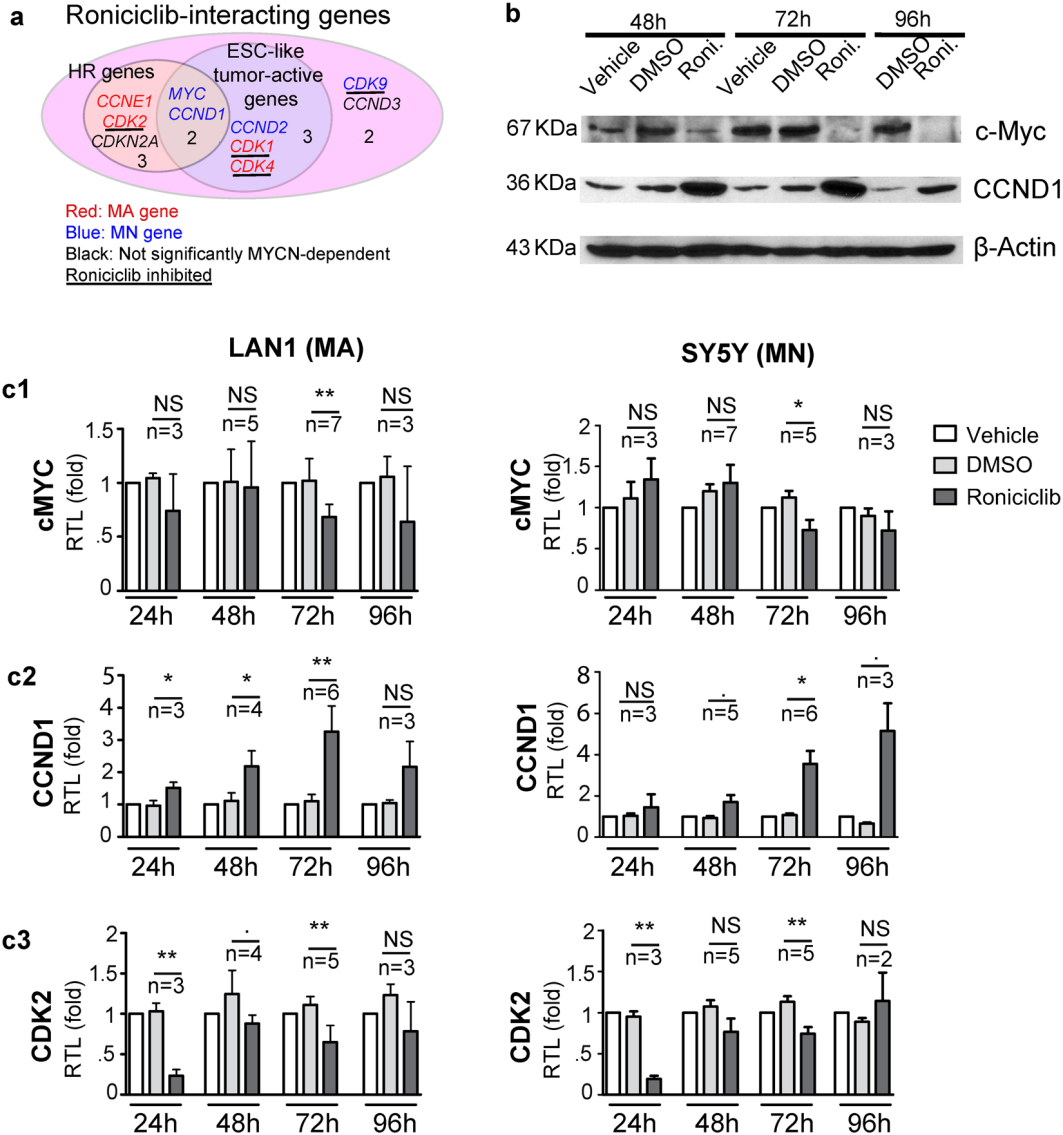
Prediction and validation of ESC-like signature genes that interact with Roniciclib, which are used as new biomarkers. (**a**) Venn diagram of the computational prediction of new biomarkers irrespective of MYCN status. Among the ten Roniciclib-interacting genes, four CDKs are inhibited and are underlined. Data resource: DGIdb. The gene symbol colour corresponds to MYCN-dependent over-expression (red: MA, blue: MN; black: NS). (**b**) Roniciclib alters c-Myc and CCND1 protein expression. The SY5Y cells were plated in 60 mm dishes one day before treatment. The cells were treated with or without vehicle control (DMSO) or Roniciclib (1 μM) for the indicated times. Then, 100 μg of protein was loaded and resolved by 10% SDS-PAGE. β-actin was used as a loading control. (**c)** Quantitative analysis of mRNA expression in a human HR-NB cell line after Roniciclib treatment for 24, 48, 72 or 96 h. The relative expression levels were normalized to the mean expression level of GAPDH. The data shown are the mean relative expression levels ± SD of replicate experiments after normalization to the non-treated control. The significance (NS > 0.1, . ≤0.1, *<0.05, **<0.01) was estimated by a two-tailed Welch’s corrected t-test that compares Roniciclib with DMSO in each scenario, followed by the number of biological replicates.

Our analyses suggested that the CDK inhibitor Roniciclib impacts both the HR-NB-specific PPI subnetwork and tumour-initiating cell mechanisms in HR-NB. To identify the drug targets, we first tested the mRNA and protein expression of potential Roniciclib targets that are over-expressed in HR-NB and belong to the ESC-like cancer signature (Fig. 5a). DGIdb (v2014) reported ten genes that interacted with the small molecule kinase inhibitor Roniciclib, five of which were over-expressed in HR-NB patients and five of which (*CCND1*, *CCND2*, *CDK1*, *CDK4* and c-Myc) belonged to the ESC-like cancer-activated signature^5^. *c-Myc* and *CCND1* were common to both the HR and ESC-like cancer signature genes and were therefore investigated.

Because c-Myc and CCND1 play triple roles as HR genes, Roniciclib targets and components of an ESC-like tumour active signature (Figs. 5a and S6A), we expected their expression to decrease in response to the potential HR-NB drug Roniciclib. However, the results of Western blotting suggested that the c-Myc protein was down-regulated, whereas the CCND1 protein was up-regulated by Roniciclib in HR-NB (Fig. 5b). Specifically, the SY5Y cells were treated with 1 μM Roniciclib at different time-points, and total lysates were analysed for the presence of c-Myc and CCND1. In accordance with the protein measurements, we observed temporal changes in the mRNA levels corresponding to Roniciclib treatment (Fig. 5c). RT-PCR analysis revealed significantly decreased c-Myc mRNA (P = 0.0043 and 0.034, Welch’s corrected two-tailed t-test), along with induced *CCND1* mRNA (P = 0.0015 and 0.011) after 72 h in both MYCN-amplified LAN1 cells and non-amplified SY5Y cells. The clear up-regulation of CCND1 after Roniciclib treat﻿ment ﻿in addition to the decrease in the protein levels of c-Myc were recapitulated by Western blotting of MCF7 breast cancer cells (Supplementary Fig. S7A), suggesting a common mechanism across tissue types. We concluded that Roniciclib-derived deregulation of c-Myc and CCND1 is independent of MYCN-amplification in HR-NB.

Additionally, we verified the changes in the protein expression of c-Myc and n-Myc in LAN1 cells (Supplementary Fig. S6). Both MYC proteins were down-regulated after 72 h of Roniciclib treatment. However, the MYCN mRNA levels showed a more restricted cell- dependent and temporal response to Roniciclib (Supplementary Fig. S6B,C). Specifically, in the LAN1 cells after 48 and 96 h of treatment, the MYCN mRNA levels were down-regulated (P = 0.029 and 0.033, n = 3). In the MYCN non-amplified SY5Y cells, the mRNA level of MYCN was up-regulated after 96 h (P = 0.028, n = 3) after exhibiting down-regulation after 48 h (P = 0.03, n = 3) of Roniciclib treatment. We concluded that Roniciclib-derived n-Myc deregulation is dependent on the MYCN status in HR-NB.

We next validated the inhibition of CDK members by Roniciclib and its potential effects on ESC-like signatures. We therefore measured changes in CDK2 and CDK4 expression in both HR-NB and breast cancer because CDK2 is a marker of ESCs, whereas CDK4 is a marker of adult stem cells^50^. Figure 5c3 shows that Roniciclib significantly down-regulated *CDK2* in both MA and MN HR-NB cells after treatment for 72 h (P = 0.019 and 0.024), but not *CDK4* (Supplementary Fig. S6B2). However, Roniciclib affected the protein and mRNA levels of both *CDK2* and *CDK4* in the MCF7 breast cancer cells after a treatment for 72 h (RT-PCR P = 0.008 and 0.022, respectively, Supplementary Fig. S7B). We concluded that Roniciclib likely inhibits an ESC-like, tumour aggressive signature in HR-NB by inhibiting CDK2.

### Roniciclib targets c-Myc-mediating tumour-initiating cell markers in HR-NB

**Figure 6:**
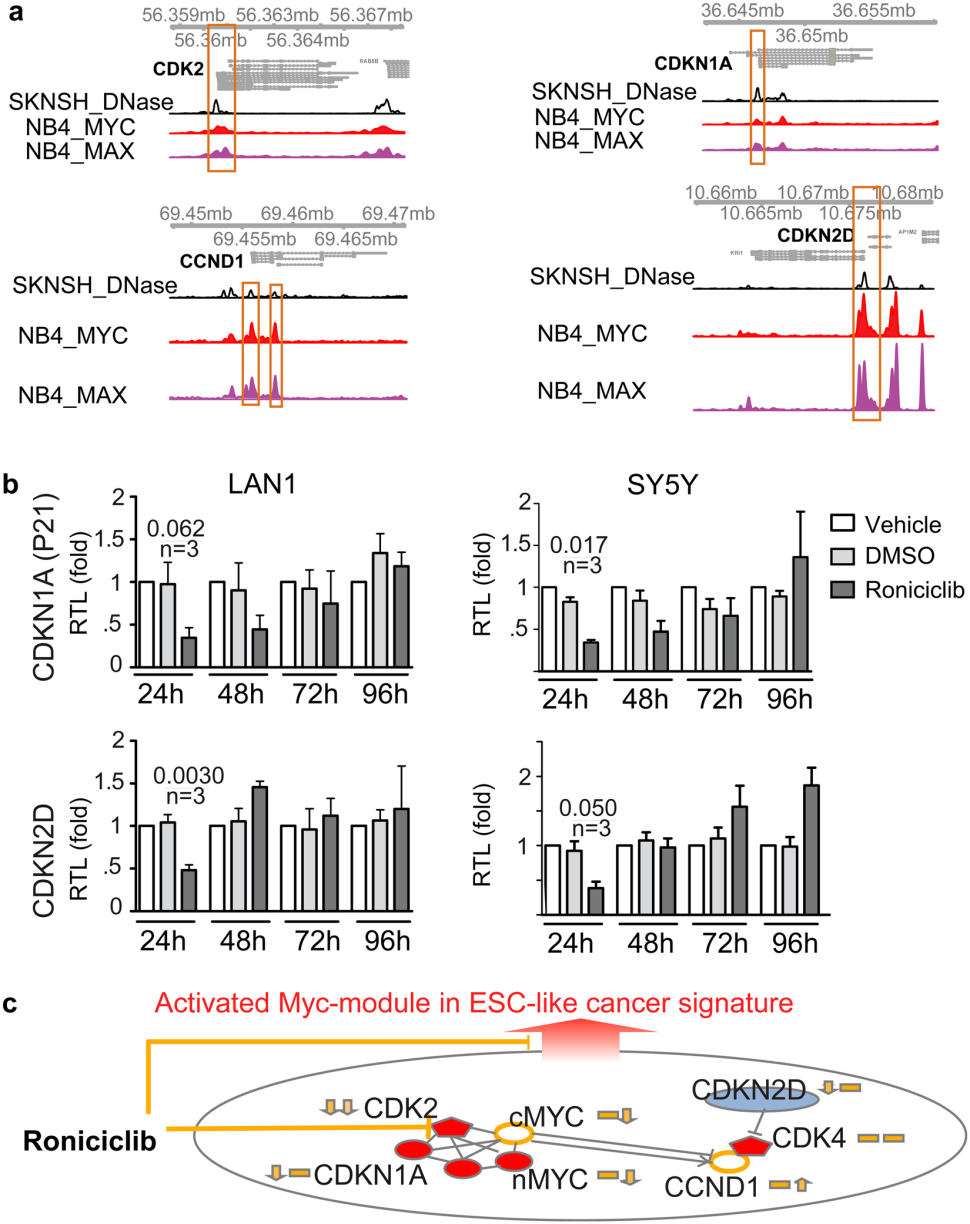
c-Myc plays a role in the Roniciclib-impacted tumour-initiating cell mechanisms in HR-NB. (**a**) Genomic view of four gene promoters showing chromatin accessibility with c-MYC/MAX occupancy (ENCODE data, hg19 assembly). The view scale of c-MYC/MAX is between 0 and 50, whereas that of DNase is between 0 and 80 in each sub-panel. (**b**) Quantitative analysis of mRNA expression in human HR-NB cells after Roniciclib treatment for 24 h. The relative expression levels were normalized to mean GAPDH expression. The data shown are the mean relative expression levels ± SD of replicate experiments after normalization to the non-treated vehicle. The p-value of a two-tailed Welch’s corrected t-test that compares Roniciclib to DMSO is given in each scenario, followed by the number of biological replicates. (**c**) Proposed model of c-Myc-regulated tumour initiating cell markers that were targeted by Roniciclib in a time-dependent manner in HR-NB. The genes indicated in red are potential neural stem cell markers that are highly expressed, and the genes indicated in blue are neural cell differentiation markers that are repressed in HR-NB compared with LR-NB. The two origin arrows along with each gene indicate the expression changes corresponding to Roniciclib treatment at an early and later phase, respectively.

We identified that the mRNA and protein levels of the HR-specific, ESC-like signature genes c-Myc and CCND1 (Fig. 5a) significantly responded to Roniciclib in HR-NB (Fig. 5b,c). Interestingly, the HR gene CDK2 is up-regulated in ENSC cells compared with differentiated adult neural cells (Fig. 4a **)**, suggesting the idea that CDK2 is a new core component of the ESC-like gene signature in HR-NB. Together, these expression changes suggest a hierarchical model of gene regulation in which Roniciclib targets the ESC-like signature in HR-NB.

c-Myc plays a essential role in this Roniciclib-impacting ESC-like signature, which is evident by the occupancy of c-Myc at both the CDK2 and CCND1 promoters in tumor (Fig. 6a). We verified the c-Myc occupancy by requiring c-Myc- and MAX-binding with chromatin accessibility at gene promoters (ENCODE data, Supplementary Methods).

In addition, we identified two new neural stem cell and differentiation markers, namely, CDKN1A and CDKN2D, which are targeted by c-Myc (Fig. 6a). CDKN1A represents the ESC-like gene signature in HR-NB, similar to CDK2 (Fig. 4a, HR genes in red in the upper right corner), and CDKN2D represents a gene signature for differentiated neural cells (Fig. 4a, LR genes in blue in the upper left corner).

To further test the effect of Roniciclib on these four c-Myc targets, we performed RT-PCR after 24–96 h of Roniciclib treatment (Figs. 5c and 6b). After 24 h, the mRNA levels of CDKN1A, CDKN2D and CDK2 decreased significantly (P-values ranging from 0.002 to 0.06, n = 3). At later time points (72 h), the CDK2 mRNA levels decreased and the CCND1 mRNA levels increased significantly (P-values ranging from 0.01 to 0.007, n ≥ 5). Similar mRNA responses to Roniciclib were observed in undifferentiated SK-N-SH ﻿cells, the parent cell line of the SY5Y cells (Supplementary Fig. S8). Thus, we concluded that Roniciclib inhibits CDK2 expression and temporally perturbs the c-Myc regulatory networks in HR-NB (Fig. 6c).

## Discussion

This study computationally extracted the importance of c-Myc from transcriptomic confluence and genetic interruptions in both HR-NB and the ESC-like cancer signatures. Coupled with *in vitro* evidence, this study further extended the emerging inhibitory effects of CDK inhibitors in HR-NB into previously uncharacterized impacts on tumour apoptosis. Furthermore, we reported the very potent therapeutic benefits of Roniciclib in targeting ESC-like signature genes in patients suffering from not only HR-NB but also other aggressive tumours in which c-Myc-regulated ESC-like signatures have been shown to contribute to oncogenesis.

The core of this study is a systems computational model evaluated in HR-NB. Three novel translational bioinformatics strategies have been applied in this study, which may be broadly applicable to computational biology research. First, we designed a topological network analysis of an ‘HR-specific, NB-associated PPI subnetwork’ and focused on its ESC-like component. This hypothesis-driven data mining enables the efficient identification of essential biomarkers for NB stemness. Second, we featured the ESC-like HR-specific signature by overlaying the neural ESC signature with the identified HR genes. To our knowledge, the computational inference of a transcriptomic ESC-like signature in HR-NB has not been reported previously, partly due to the difficulty in identifying cell surface markers of ESCs in NB^51^. Third, we successfully stratified patients using the RXA-GSP algorithm, which quantifies the systematic imbalance between poor-outcome and good-outcome components in individuals^47^. The RXA-GSP method improves the robustness of previous multi-gene prognostic models that lack standards of measurement across various studies, assays, and diagnostic platforms^52^. The future combination of these strategies with other approaches will facilitate the computational characterization of tumour-initiating cells and urgently needed precise therapy. Furthermore, these approaches will have broad relevance to the evaluation of high-risk tumour states with previously uncharacterized genomic features.

The identification of additional biomarkers for high-risk patients without MYCN amplification is of clinical importance. All NB patients with amplified MYCN copy numbers are considered high risk^8, 9^. However, outcomes are highly variable within this group^53, 54^, and few prognostic biomarkers are available for MYCN-non-amplified patients. In the present study, we showed that n-Myc and its homologue c-Myc share common targets in a c-Myc interactome related to high-risk neuroblastoma. We further revealed a novel pharmacological role and drug-targets as biomarkers that are independent of MYCN status. These findings shed new light on the precise diagnosis and treatment of all HR-NB, specifically in cases without MYCN amplification, to ensure that therapy is appropriately tailored to each individual.

Recently, CDK inhibitors have emerged as a rational pharmacological approach to inhibit the transcription of critical MYC-regulated oncogenic, metastatic, and stem-cell programs^55, 56^. Hyper-activation of CDK regulatory pathways has been observed in multiple human cancers, including NB^57^. Among the pharmacological inhibitors of CDK, Roniciclib is currently in clinical trials for the treatment of small cell lung cancers or advanced solid tumours that are refractory to standard therapy (ClinicalTrials.gov identifiers: NCT02522910, NCT01188252, NCT02390154, NCT01573338, NCT02161419 and NCT02457351). Although CDKs have emerged as a novel therapeutic option for HR-NB^58–60^, we demonstrated the exciting pharmacological potential of Roniciclib based on its ability to induce tumour cell apoptosis and growth arrest in HR-NB. Furthermore, we showed that Roniciclib exerts potent therapeutic effects by targeting the c-Myc subnetwork in HR-NB, which has the potential to open new avenues for the development of stemness-targeting medicines and treatment-resistant cancer therapies.

c-Myc, which is down-regulated by Roniciclib, targets the HR and ESC-like marker gene CCND1 in HR-NB. Therefore, the induced expression of CCND1 by Roniciclib treatment is unexpected. One potential explanation is that CCND1, unlike other cyclin family members (A and E) whose expression is elevated by c-Myc, can be both activated and repressed by c-Myc^61, 62^. In this case, Roniciclib treatment down-regulates c-Myc in tumour cells and relieves the suppression of CCND1 by c-Myc. However, Roniciclib treatment for 24–48 h altered the CDK2 and CCND1 mRNA levels but did not down-regulate the c-Myc mRNA levels in both cell lines (Fig. 5c), suggesting the existence of another CCND1 regulator that responds to Roniciclib. Given that CDK2 doesn’t bind to CCND1^63^, the deregulation of cyclin D1 could be caused by a hierarchical model of gene regulation in which Roniciclib targets CDK2 and c-Myc.

The primary goal of the systematic informatics and biological approaches applied here is the translation of genomic and transcriptomic data into molecular mechanisms underlying high-risk tumours. This work supports a model in which c-Myc regulates an ESC-like cancer-activated signature that primarily corresponds to tumour types with poor clinical outcomes^5, 64, 65^. Our data added a new paediatric tumour type, HR-NB, to this model, suggesting that targeting stem cell-like signatures may ultimately affect chemoresistant tumours. Furthermore, our study revealed a previously unsuspected complexity between the associations of CDK inhibitors with MYC regulation. Specifically, Roniciclib selectively inhibits CDK2, not CDK4, in HR-NB but not in breast cancer, which results in new hypotheses regarding both CDK inhibition and cancer stem cell-like signatures. Furthermore, the prognostic power of CDKN3, a canonical CDK2 repressor, suggests its novel role as a neural ESC marker. Finally, our new observations suggest that Roniciclib interferes with multiple genes in a time-dependent manner to affect the c-Myc network in NB cells. Roniciclib acts at an early phase (24 h) by targeting CDK2 and cell cycle arrest, leading to cell senescence^42^. During later phases of treatment (72 h), Roniciclib activates Caspase-3 to initiate cell apoptosis. However, these hypotheses require further investigation to elucidate the underlying fundamental biological mechanisms.

## Materials and Methods

### Collection of transcriptomic profiles

Transcriptomic data pertaining to patients with neuroblastoma were collected from the public databases GEO and ArrayExpress (Supplementary Table S1).

### MA-dependently expressed gene signatures

To identify a MA-dependently expressed gene signature, we compared the transcriptomic profiles of patients with *MYCN*-amplification (>10 copies, **MA**) to those with 2 copies (**MN**). To increase the statistical power, we pooled samples measured on the same platform after normalization and corrected the batch effects^66^ using an empirical Bayes approach called COMBAT^27^. The identified MA- and MN-signature genes were significant at an FDR of 0.001, presenting the same expression orientation across the tested cohorts.

### Transcriptional HR and LR signatures

Patients without MYCN amplification may be at high risk but lack clinical biomarkers. Therefore, we focused on patients with normal MYCN copy numbers. We used the limma test to identify genes that were differentially expressed in at least one studied cohort (FDR < 0.05, fold-change >1.3, 1.5 or 1.8 (Supplementary Table S1), and each gene prioritized the leading 2,000 or less differentially expressed genes in each cohort). We hereafter refer to the up-regulated genes in HR-NB patients with MN as the “**HR genes** ” and the genes up-regulated in LR-NB patients with MN as the “**LR genes**”.

### A collection of genes with genomic variants

Genes with somatic or germline mutations in exons were collected from publications and the National Cancer Institute’s Therapeutically Applicable Research to Generate Effective Treatments (TARGET). We refer to both types of variants as “genomic variants” hereafter, and focus on the variation identified in HR-NB patients^67–70^. This collection resulted in a set of 987 genes with recurrent HR-NB-associated variants and a set of 197 genes with validated somatic mutations.

### Functional enrichment analysis

We reported the biological process (BP) terms (GO.db 2.14.0) with an odds ratio larger than 2 and an FD <0.05 that contained at least five signature genes using a background-adjusted Fisher’s exact test (FET)^71^. We conducted a drug-gene enrichment analysis against 197 genes with verified genomic variants and 987 genes with recurrent somatic mutations in HR-NB based on the DGIdb database^41^ (v1.72). Significance was recognized at an FDR <0.05 or 0.01 with at least five interacting genes by performing a background-adjusted FET^71^.

### NB-associated subnetwork

This subnetwork consisted of transcriptionally or genetically interrupted genes among HR patients and was constructed in two steps: 1) We pooled the 4,425 identified MA and MN genes and 1097 genes with verified or recurrent somatic mutations in HR-NB. 2) We queried the STRING (v10) PPI database and obtained a subnetwork of 4,460 connected proteins with a combined STRING confidence (score) of 0.7 or higher.

### HR-NB-specific PPI subnetwork

We performed a topological network analysis of the PPI network by querying the STRING^33^ interactome databases (Fig. 2d) and then abstracted two subnetworks. One subnetwork consisted of the HR genes and thus represented unfavourable outcomes and potential oncogenesis effects in HR-NB (termed the “**HR-NB-specific PPI subnetwork**”). As a control, the other subnetwork consisted of the LR genes and thus represented favourable outcomes and potential tumour-suppressor effects. Nodes in the “HR-NB-specific” PPI sub-network were components that belong to, or directly interact with HR but not LR gene components.

### Network hub and bottleneck

We then analysed the betweenness centrality (the number of the “shortest paths” traversing a network vertex) and the connection degree in each subnetwork. We defined “bottleneck” proteins as those that were in the top 2% in terms of betweenness and defined “hub” proteins as those that had the 2% highest number of neighbours^25^. Hubs and bottlenecks can occur independently. The Supplementary Methods describe the estimation of empirical significance.

### I-score indicating patient survival

We developed an individualized algorithm, termed the relative expression of a gene-set pair (GSP), (﻿Equations 1 and 2) to choose t﻿he GSPs ﻿from N possible co﻿mbinations that can successfully stratify patients into prognostic groups^47^. In this study, a prognostic indicator (the I-score) was defined as the ratio of the expression of a ‘set of poor-outcome markers’ (Sp) to the expression of a set of good-outcome markers (Sg). Therefore, a high indicator is expected to indicate an unfavourable outcome. Let the cardinality (count of genes) of each gene-set S be |S|. Considering combinations of the three poor-outcome markers and three good-outcome markers herein, 49 GSPs are possible using the pooled transcriptomic profiles of 295 patients from six datasets (Supplementary Table S1) (Equation 3). Note that some of the training dataset did not measure the marker CDK19, resulting in the exclusion of seven potential indexes that consisted of CDK19.

1 $${\rm{I}}={\rm{median}}({\rm{HR}}\text{\_}{\rm{signature}})-{\rm{median}}({\rm{LR}}\text{\_}{\rm{signature}})$$

2 $$N=\sum _{i={\rm{2}}}^{| MA\text{\_}hi| }(\begin{matrix}| MA\text{\_}hi| \\ i\end{matrix})\times \sum _{j={\rm{2}}}^{| MN\text{\_}hi| }(\begin{matrix}| MN\text{\_}hi| \\ j\end{matrix})$$

3 $${N}^{\ast }=\sum _{i=1}^{|Sg|}(\begin{matrix}|Sg|\\ i\end{matrix})\times \sum _{j=1}^{|Sp|}(\begin{matrix}|Sp|\\ j\end{matrix})$$

We tested the association of this indicator with event-free survival outcomes as a dichotomous stratification (positive vs. negative indicator) with a Kaplan-Meier analysis (log-rank test) using R with the survival package. The event-free survival information was derived from the original publications. First, we evaluated the prognosis of MYCN-amplification and the prognosis of our I-score for patients in the six signature-training datasets as a control. We then validated the prognosis for newly diagnosed patients from an independent dataset, E-MTAB-1781^48^.

### Tumour cell lines

We selected the SY5Y, LAN1 and SK-N-SH (ATCC HTB-11) cell lines due to their different genomic characteristics: LAN1 exhibits MYCN amplification, whereas SY5Y and SK-N-SH cells do not ﻿exhibit this amplification. Note that SY5Y, a human cell line derived from SK-N-SH cells, is widely used in scientific research. The SY5Y and LAN1 cell lines were kindly provided by the laboratory of Dr. Susan Cohn at the University of Chicago. The SK-N-SH (ATCC HTB-11) cell line and the breast cancer cell line MCF7 (ATCC HTB-22) were purchased from the American Type Culture Collection (ATCC).

### Roniciclib treatment

Roniciclib was purchased from AdooQ Bioscience (Irvine, CA, USA), dissolved in DMSO at 0.5 mM and 1 mM and stored for later use. Tumour cells were cultured in RPMI medium supplemented with 10% fetal bovine serum and 1% penicillin/streptomycin and maintained at 37 °C in 5% CO_2_. For drug treatment, the cells were seeded the day before the drug treatment and then treated with 1 μM Roniciclib for 24–96 h, with DMSO (Sigma), or with nothing as the vehicle control.

### Evaluation of cell morphology

Cells were plated at 40% confluence and allowed to grow for 12–18 h before being treated with Roniciclib (1 μM) for 48 h to 72 h. Cell morphology was then examined, and cell images were obtained using the 20X objective of a Leica DM IRB microscope.

### Quantification of gene expression levels

We performed quantitative Real-time PCR (qRT–PCR) analysis. Because the IC50 of Roniciclib for cell proliferation is approximately 1 μM (Supplementary Fig. S4), we treated cells with 1 μM of Roniciclib for the qRT-PCR experiments. The comparative method (ΔΔCt) was used to calculate relative expression. Non-treated cells were used as a reference, and GAPDH was used as an internal control. The relative mRNA levels of at least three biological replicates of different genotypes were compared based on the ΔΔCt values using an unpaired Student’s t-test with Welch’s correction; P < 0.05 was considered significant. Primer sequences and other technical details are described elsewhere in the Supplementary Methods.

### Western blotting

The SY5Y and MCF7 cells were treated with the indicated reagents and collected at different time points (48 and 72 h after treatments). Primary antibodies against c-Myc (C-19) (sc-788), Cyclin D 1/2 (clone 5D4) (Millipore: MAB3658), and β-Actin (C-4) (sc-47778) were purchased from Santa Cruz Biotechnology or Millipore.

## Electronic supplementary material

Supplementary Information
