# Supplementary material for: A c-Myc-regulated stem cell-like signature in high-risk neuroblastoma: A systematic discovery (Target neuroblastoma ESC-like signature)
Source: Sci Rep. 2017 Mar 3;7:41. doi: 10.1038/s41598-017-00122-x (PMC5427913; doi:10.1038/s41598-017-00122-x)
Supplement: Supplementary file 1 — Supplementary Information [file 41598_2017_122_MOESM1_ESM.pdf]

A c-Myc regulated stem cell-like signature in high-risk neuroblastoma: a systematic discovery

(Target neuroblastoma ESC-like signature)

Xinan (Holly) Yang*, Fangming Tang, Jisu Shin, John M Cunningham*
Section of Hematology/Oncology, Dept of Pediatrics, U of Chicago, Chicago, IL 60637, USA

Supplementary Methods

1. MYCN-dependently expressed gene signature via meta-analysis of expression profiles

We collected a total of 556 primary patients from four independent studies to derive this signature. Raw gene expression profile data (Affymetrix.CEL files or Agilent.txt files) and the clinical information for patients were retrieved from seven datasets at different GEO[1] or ArrayExpress[2], comprised of 325 patients of advanced stage (stages 3 and 4) neuroblastoma with outcome records (**Supplementary Table 1**). Expression measurements were transformed to the log2 level if they were not originally done so by the authors. The downloaded Affymetrix exon arrays were processed using the Bioconductor *aroma.affymetrix* package for each dataset[3]. For the custom designed Agilent arrays, we re-annotated the probes to the human genome assembly hg37 by integrating the annotations derived by the BLAST algorithm and that by the DAVID online annotation tools[4]. To increase statistical power, we pooled samples measured on the same platform into a big dataset after normalization[5]. COMBAT[6] was applied to adjust the batch effects.

For differential expression analysis, we performed variance-stabilizing transformation with the Linear Models for Microarray Data (limma) method on the two pooled datasets, respectively. We selected the probe with the lowest p-value to represent the transcriptional change of its designed gene if multiple probes were mapped to the same gene on the array. We then applied Stouffer meta-analysis to combine the linear model resultant statistics into jointed p-values. For multiple testing correction, we considered

the genes with probes meeting a false discovery rate (FDR) less than 0.001 with agreement on the orientation of deregulation across all datasets for downstream analyses. Additionally, we included significant genes but only measured by one platform for this meta-analysis to maximum the candidate gene signatures.

Finally, we split the identified MYCN-dependently expressed genes into two sets of gene signatures. The MA genes were higher-expressed in MYCN-amplified (MA) patients than in MYCN-non-amplified (MN) patients with high-risk neuroblastoma (HR-NB), while the MN genes were low-expressed in MA patients with HR-NB (i.e., higher-expressed in MN patients) than in MA patients. Note that we are interested in the collective effects of the MA genes compared to the MN genes rather than the expression of any individual genes.

2. Define transcriptional "HR genes" and "LR genes" from MN patients

To identify the genes overexpressed in high-risk patients, we analysed six cohorts collected from nine gene expression data-sets containing patients with normal MYCN copy number. Since all patients with MYCN amplification will be categorized into the high-risk group, we focused on the patients with normal MYCN copy number to make the comparison between high-risk and low-risk groups. Again, we combined data-sets with a small sample size (< 30) after using COMBAT[6] to adjust the batch effects. We used the limma test to identify genes that showed significance in at least one studied cohort (FDR<0.05, fold change>1.3, 1.5 or 1.8 (**Supplementary Table S1**) and prioritized up to 2000 differentially expressed genes in each cohort. When multiple probesets were designed for one gene in a platform, we kept the one with the lowest p-value and then adjusted the significance of multiple tests among all genes. We excluded genes showing conflicting orientations of fold changes across multiple cohorts, and calculated the average fold change if a gene presenting the same statistically significant orientation across multiple cohorts.

3. Three well-known MYCN-independent prognostic signatures in HR-NB[7-10]

Asgharzadeh et al. reported a 55-probeset (52-gene) signature stratifying *MYCN* non-amplified stage 4 patients with poor outcome from those with a high survival probability[7]. Comparing the mRNA expression patterns of high-risk patients with those expression patterns of low-risk patients, Vermeulen et al. identified 59 prognostic genes for NB in at least two of seven cohorts[8]. A year later, the same group purified these 59 genes into a 42-gene signature to be robustly prognostic in independent primary NB, even in HR-NB[9]. Additionally, Valentijn et al. identified a 157-gene signature of *MYCN* targets by shRNA-mediated silencing *MYCN* in NB cells, and showed that its prediction of poor prognosis to be more powerful than MYCN-amplification or the mRNA expression of MYCN[10].

4. Collecting genomic variations in high-risk NB

Genes with somatic mutations in exons were collected from two publications including the study of the National Cancer Institute's Therapeutically Applicable Research to Generate Effective Treatments (TARGET). From 240 patients with HR-NB, a recent study identified a spectrum of 5291 candidate somatic mutations in the coding regions of 3960 genes. Among them, 268 potential RH-NB causing mutations of 195 genes have been verified[11]. Another study of 87 untreated primary NB tumours of all stages, including 48 patients with HR-NB, revealed 157 genes harbouring verified somatic variations with a somatic score larger than 0.1[12]. From the first study, we collected 182 genes that harbour somatic missense-mutations with evidence, as a coding region missense mutation will mostly impact the gene function. To maximum the research candidates, we also collected the patients with HR-NB from the second study if the they harboured the verified somatic mutations. Additionally, we included *LMO1* with the germline variant associated with HR-NB for a following query for prognostic signature (reviewed by [13]) because *LMO1* genotypes are significantly associated with metastatic and high-risk status in HR-NB[14].

5. Regulatory network analysis

Gene members of the over-represented genesets were linked if their protein products interact in the STRING (v10)[15] PPI databases. We used *Cytoscape* to visualize the network and the Bioconductor *igraph* package to calculate the network degree. The network hubs were identified by both empirical and theoretical tests. Specifically, to evaluate the empirical degree significance, we randomly sampled the same number of gene symbols from the STRING database (STRINGdb) and calculated the empirical significance after B=2000 rounds of simulations, which was calculated by P = # of (simulated degree > observed degree)/B. Meanwhile, we calculated the theoretical significance for each protein in the network using the Fisher's Exact test (FET). This theoretical test compared the observed network-degree in the NB-associated subnetwork to the background degree in the STRING database. The significance was identified with an odds ratio larger than 2 and a p-value less than 1e-5.

6. MYC occupancy at gene promoters in HR-NB

The following data were retrieved from ENCODE (hg19 assembly):

DNase-seq in the SK-N-SH cells: wgEncodeOpenChromDnaseSknshBaseOverlapSignal.bigWig

ChIP-seq for cMyc in NB4 cells: wgEncodeSydhTfbsNb4CmycStdSig.bigWig

ChIP-seq for MAX in NB4 cells: wgEncodeSydhTfbsNb4MaxStdSig.bigWig

Genomic view were generated using Bioconductor packages Gviz and GenomicRAnges, and the gene coordinates were retrieved from the UCSC genome assembly hg19.

7. Cell proliferation assay

To monitor the growth rate of cell population in growing population, cell proliferation was assessed by

the 3-(4,5-dimethylthiozol-2-yl)-5-(3-carboxymethoxyphenyl)-2-(4-sulfophenyl)-2H-tetrazolium, inner salt (MTS) assay (Promega Corporation, Madison, WI) according to the manufacturer's instructions. Cells were seeded in 96-well plates (30,000 cells/well) in RPMI-1640 medium containing 10% FBS. The absorbance of the formazan at 490 nm were measured directly from the 96-well plates. Cells (100 μl/well) were seeded at seeding densities of 3x104 into 96 well microtitre plates and allowed to adhere for 16 h. Cells were treated with different dose of Roniciclib in 100 μl solution. To monitor the conversion of MTS to formazan, cells was assessed on a daily basis by adding 20 μl of MTS. Following 1 h incubation period with MTS, the absorbance at 490 nm was recorded using an ELISA plate reader. The background was at zero cells/well, and DMSO was used as vehicle control.

8. **Detection of apoptosis**

To determine the number of dead cells in population after Roniciclib treatment, active caspase 3 was detected using the Caspase-3 DEVD-R110 Fluorometric HTS Assay Kit (Biotium, Fremont, CA). The substrate was completely hydrolyzed by the enzyme in two successive steps. Cleavage of the first DEVD peptide resulted in the monopeptide Ac-DEVD-R110 intermediate, which had absorption and emission wave-lengths similar to those of R110 ($\lambda_{abs}/\lambda_{abs}$= 496/520 nm). Hydrolysis of the second DEVD peptide released the dye R110, leading to a substantial fluorescence increase. R110 was used for generating a standard curve to calculate the amount of the substrate conversion.

The SY5Y cells were placed in 100 μl culture medium per well of a black 96-well plate in the same cell number as described for the cell viability assay. Cells were treated by different concentration of Roniciclib to induce apoptosis. Caspase-3 activity was detected by adding 100 ul of assay buffer to 100 ul cells in culture medium in each well at different time points. The plate was incubated at 37°C for 60 minutes and fluorescence with 470 nm excitation and 520 nm emission.

9. RNA extraction and Real Time – Quantitative PCR

Total RNA was extracted from a maximum of 2-3 million cells per sample using TRIzoL Reagent (Ambion, Invitrogen) according to the manufacturer's instructions. After RNA extraction, equal amounts of total RNA from different cell lines (1 ug) were retro-transcribed using the SuperScript III First-Strand Synthesis System for RT-PCR (Invitrogen, Carlsbad, CA, USA) in the conditions described by the manufacturer.

All Real-time reactions were carried out with Random Hexamers. Subsequently, the obtained cDNA was used as template for PCR using the Power SYBR Green PCR Master Mix System (Applied biosystems, Warrington WA1 4SR, UK). Primers specific for human *MYCN, cMYC, CDK2, CDK4, CCND1, CDKN1A,* and *CDKN2D* cDNA were used for amplification and human GAPDH for internal control. All assays were carried out in a 96-well format. Real-time fluorescent detection of PCR products was performed with a 7500 Standard Real-Time PCR System (Applied Biosystems) using the following thermocycling conditions: 1 cycle of 50°C for 2 min and 95°C for 10 min; 40 cycles of 95°C for 15 s, and 60°C for 1 min; 95°C for 15 s and 60°C for 1 min; 95°C for 30 min and 60°C for 15 min.

Primer sequences were:

GAPDH-F: 5' GGAGTCCACTGGCGTCTTC 3'

GAPDH-R: 5'ATCTTGAGGCTGTTGTCATACTTC 3'

MYCN-F: 5' GACCACAAGGCCCTCAGTACC3'

MYCN-R: 5' TGACCACGTCGATTTCTTCCT 3

cMYC-F: 5' TGAGGAGACACCGCCCAC 3'

cMYC-R: 5' CAACATCGATTTCTTCCTCATCTTC 3'

CCND1-F: 5'- CCGAGAAGCTGTGCATCTACA-3'

CCND1-R: 5'-AGGTTCCACTTGAGCTTGTTCAC-3'

CDK2-F: 5'-ATGGGTGTAAGTACGAACAGG-3'

CDK2-R: 5'-TTCTGCCATTCTCATCGG-3'

CDK4-F: 5'-GGCAGAGATTCGCTTGTG-3'

CDK4-R: 5'-CTGTGGACATGTGGAGTGTTG-3'

CDKN1A-F: 5'-GCCTTAGCCCTCACTCTGTG-3'

CDKN1A-R: 5'-AGCTGGCCTTAGAGGTGACA-3'

CDKN2D-F: 5'-TCACACTGCTGTGGTCAGCTTT-3'

CDKN2D-R: 5'-AGGATGTCCACGAGGTCCTGA-3'

10. Western blotting

SY5Y and MCF7 cells were treated with indicated reagents and collected at different time points (48, 72 and 96 hours after the treatments). Primary antibodies against c-Myc (C-19) (sc-788), Cyclin D 1/2 (clone 5D4) (Millipore: MAB3658), and β-Actin antibody (C-4) (sc-47778) were purchased from Santa Cruz Biotechnology or Millipore. The human n-Myc polyclonal antibody was purchased from Thermo Fisher (Product # PA5-17403). Cells were lyzed in cold RIPA buffer with protease inhibitors and centrifuged (13,000 rpm for 15min at 4°C) and the supernatant was used for blotting. The lysates were boiled in loading buffer with sodium dodecyl sulphate (SDS) for 5 min. The supernatant was then separated by SDS–polyacrylamide gel electrophoresis (SDS–PAGE), transferred to nitrocellulose membranes, and immunoblotted with primary antibodies and visualized with horse- radish peroxidase-coupled anti-mouse or -rabbit immunoglobulin IgG antibodies using the SuperSignal Peroxidase and Luminol/Enhancer solution (Thermo Scientific).

Reference for the Supplementary Methods

1 Barrett, T. *et al.* NCBI GEO: archive for functional genomics data sets--10 years on. *Nucleic Acids Res* **39**, D1005-1010, doi:10.1093/nar/gkq1184 (2011).

2 Parkinson, H. *et al.* ArrayExpress update--from an archive of functional genomics experiments to the atlas of gene expression. *Nucleic Acids Res* **37**, D868-872, doi:10.1093/nar/gkn889 (2009).

3 Bengtsson, H. & Hossjer, O. Methodological study of affine transformations of gene expression data with proposed robust non-parametric multi-dimensional normalization method. *BMC Bioinformatics* **7**, 100, doi:10.1186/1471-2105-7-100 (2006).

4 Dennis, G., Jr. *et al.* DAVID: Database for Annotation, Visualization, and Integrated Discovery. *Genome Biol* **4**, P3 (2003).

5 Cohn, L. D. & Becker, B. J. How meta-analysis increases statistical power. *Psychol Methods* **8**, 243-253, doi:10.1037/1082-989X.8.3.243 (2003).

6 Leek, J. T. *et al.* Tackling the widespread and critical impact of batch effects in high-throughput data. *Nat Rev Genet* **11**, 733-739, doi:10.1038/nrg2825 (2010).

7 Asgharzadeh, S. *et al.* Prognostic significance of gene expression profiles of metastatic neuroblastomas lacking MYCN gene amplification. *J Natl Cancer Inst* **98**, 1193-1203, doi:10.1093/jnci/djj330 (2006).

8 Vermeulen, J. *et al.* Predicting outcomes for children with neuroblastoma using a multigene-expression signature: a retrospective SIOPEN/COG/GPOH study. *Lancet Oncol* **10**, 663-671, doi:10.1016/S1470-2045(09)70154-8 (2009).

9 De Preter, K. *et al.* Accurate outcome prediction in neuroblastoma across independent data sets using a multigene signature. *Clin Cancer Res* **16**, 1532-1541, doi:10.1158/1078-0432.CCR-09-2607 (2010).

10 Valentijn, L. J. *et al.* Functional MYCN signature predicts outcome of neuroblastoma irrespective of MYCN amplification. *Proc Natl Acad Sci U S A* **109**, 19190-19195, doi:10.1073/pnas.1208215109 (2012).

11 Pugh, T. J. *et al.* The genetic landscape of high-risk neuroblastoma. *Nat Genet* **45**, 279-284, doi:10.1038/ng.2529 (2013).

12 Wang, K. *et al.* Integrative genomics identifies LMO1 as a neuroblastoma oncogene. *Nature* **469**, 216-220, doi:10.1038/nature09609 (2011).

13 Capasso, M. *et al.* Replication of GWAS-identified neuroblastoma risk loci strengthens the role of BARD1 and affirms the cumulative effect of genetic variations on disease susceptibility. *Carcinogenesis* **34**, 605-611, doi:10.1093/carcin/bgs380 (2013).

14 Deyell, R. J. & Attiyeh, E. F. Advances in the understanding of constitutional and somatic genomic alterations in neuroblastoma. *Cancer Genet* **204**, 113-121, doi:10.1016/j.cancergen.2011.03.001 (2011).

15 von Mering, C. *et al.* STRING: known and predicted protein-protein associations, integrated and transferred across organisms. *Nucleic Acids Res* **33**, D433-437, doi:10.1093/nar/gki005 (2005).

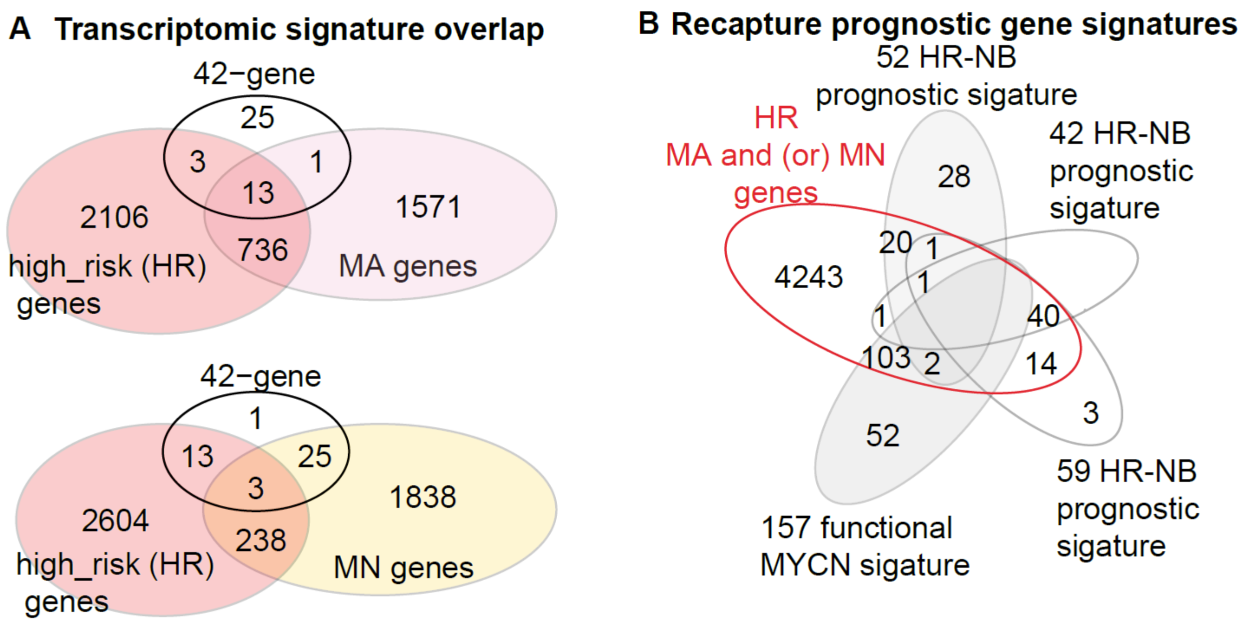

C

	52-gene signature	42-gene signature	59-gene signature	157-gene signature
# of overlap	24	42	56	105
FET p-value	0.0038	<2e-16	<2e-16	<2e-16
Odds ratio	2.8	Inf	62.2	6.8

Figure S1. The associations between prognosis, genomic variants, and transcriptional MYCN-association in HR-NB.

A) Venn diagram of overlaps among the HR genes, the prognostic 42-gene classifier, and the MA genes (top sub-panel) or the MN genes (bottom sub-panel). By comparing these two sub-panels, we show that HR genes recapture both MA and MN genes and are significantly enriched for the MA genes. However, from the viewpoint of the prognostic 42-gene classifier, it significantly recaptures both MA and MN genes and shows preference for the MN genes.

B) Due to the heterogeneity of NB, no one gene was recaptured among all three well-known prognostic signatures (the 52-, 42- and 157-gene signatures), and only three genes were recaptured by two prognostic signatures. However, our MA and MN genes recapture the majority of these three well-known prognostic signatures. Note that the 42-gene signature is further derived from the 59-gene signature.

C) The results of Fisher's exact tested (FET) for the overlap among the MA-dependently expressed signature (the merger of MA and MN genes) and four published HR-NB prognostic signatures. A background of 19,000 measured human gene symbols are used for the tests.

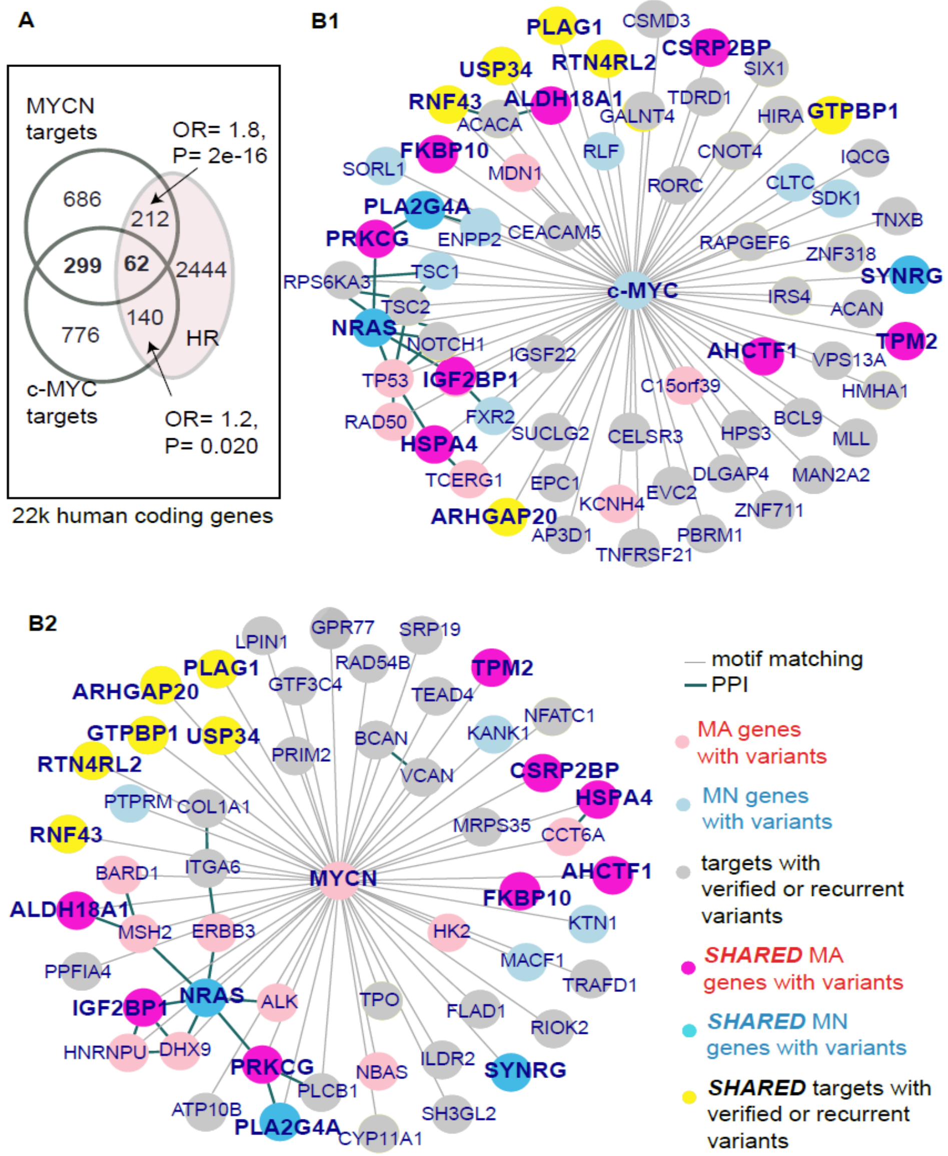

Figure S2. MYC's oncogenicity can be further enhanced by collaborations with other oncogenes such as a RAS mutant or activation.

A) Venn diagram of HR-signature genes and the regulatory targets of MYCN and c-Myc. 1259 previously published MYCN targets (that either contain MYCN-binding motif or co-expression of MYCN in neuroblastoma or small cell lung cancer cells) are significantly enriched with 1277 c-Myc targets containing c-Myc binding motifs (299+62=361 overlaps, P<2e-16, OR=8.2). Note that MYCN is over-expressed in MA HR-NB and c-Myc is over-expressed in MN HR-NB, thus showing different node colors.

B) The network view of the subset of MYCN targets and c-Myc targets, respectively, that carry verified or recurrent somatic mutations. The grey line corresponds to protein-DNA binding based on sequences, and the green line corresponds to protein-protein interaction based on knowledge (STRING v9.05). Node colour corresponds to MYCN-dependent over-expression (red or wine: MA, blue or light blue: MN; grey or yellow: NS). Notably, NRAS is co-targeted by both MYCN and c-Myc.

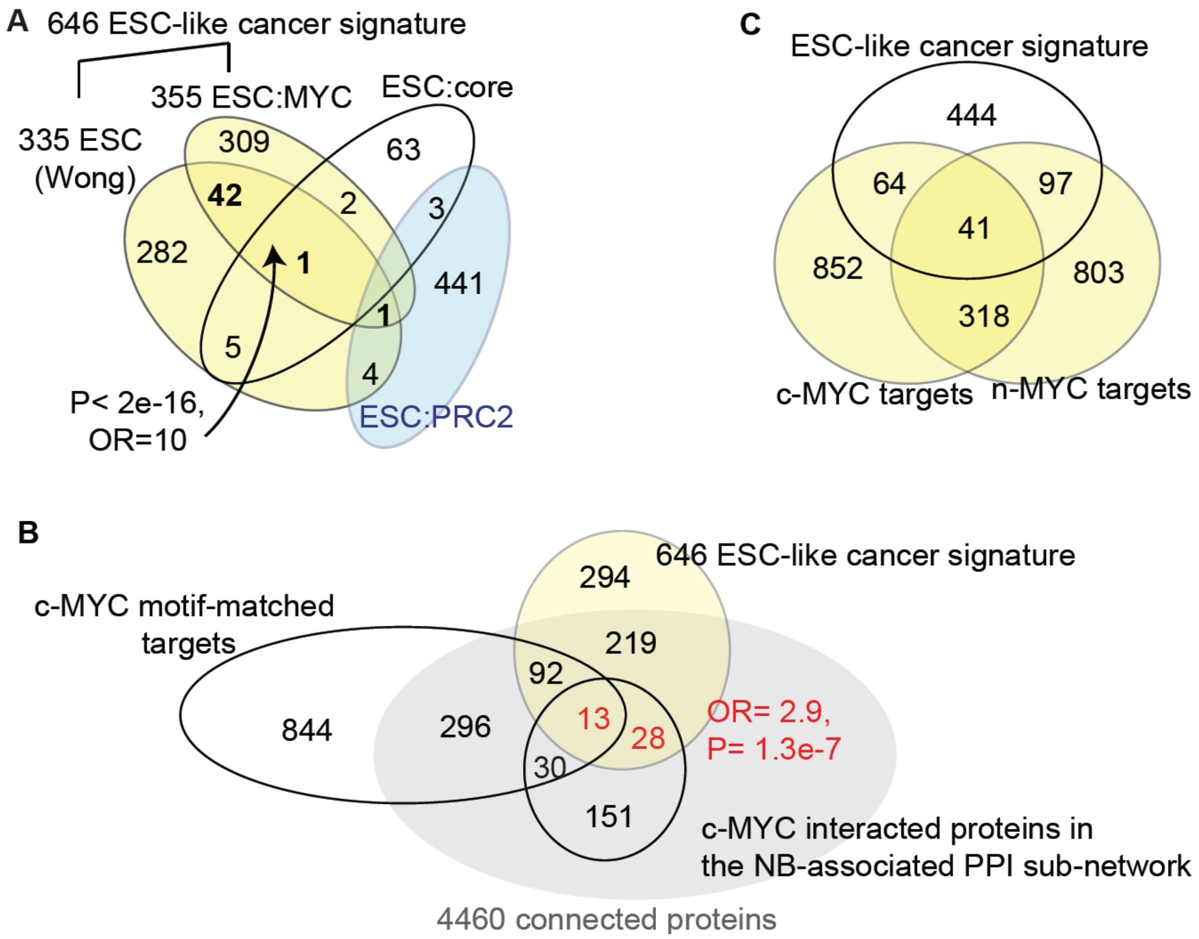

Figure S3. Association between c-Myc and ESC-like gene signature.

A) Venn diagram of the ESC-like signatures. We defined an "ESC-like cancer signature" by pooling two sets of cancer-active ESC-like signatures (highlighted in yellow): the 335 cancer-activated, ESC-like signature genes defined by Wong and colleagues (Wong, Liu et al. 2008) and the c-Myc-dependent ESC-like 355-gene module defined by Kim and colleagues (Kim, Woo et al. 2010). Wong's ESC-like cancer-activated signature is expressed in both ESCs and adult tissue stem cells and is activated in diverse human epithelial cancers. Kim et al then further sub-divided the ESC-like signature into three modules, including c-Myc-related factors (a MYC module). The MYC module reported by Kim and colleagues significantly over-represents the cancer-activated ESC-like module in Wong's study (P<2e-16, OR=10), but not the other two units. PRC: Polycomb complex factors.

B) A Venn diagram of c-Myc neighbouring proteins and hubs in the neuroblastoma-associated PPI subnetwork, as well as the ESC-like cancer signature. c-Myc interacting proteins significantly overlap with the 646 ESC-like cancer signatures (P=1.3e-7, OR=2.9), as 41 out of its 221 c-Myc neighbours in the NB-associated PPI were defined as ESC-like cancer signatures. Statistics were performed among the 4460 connected proteins with a combined STRING confidence of 0.7 or higher.

C) A Venn diagram of MYC targets and the ESC-like cancer-activated gene signature. Significance of pair-wise overlap exists. Both c-Myc and n-Myc significantly target the ESC-like cancer signature genes (FET P<2e-16, OR=3 and 5, respectively).

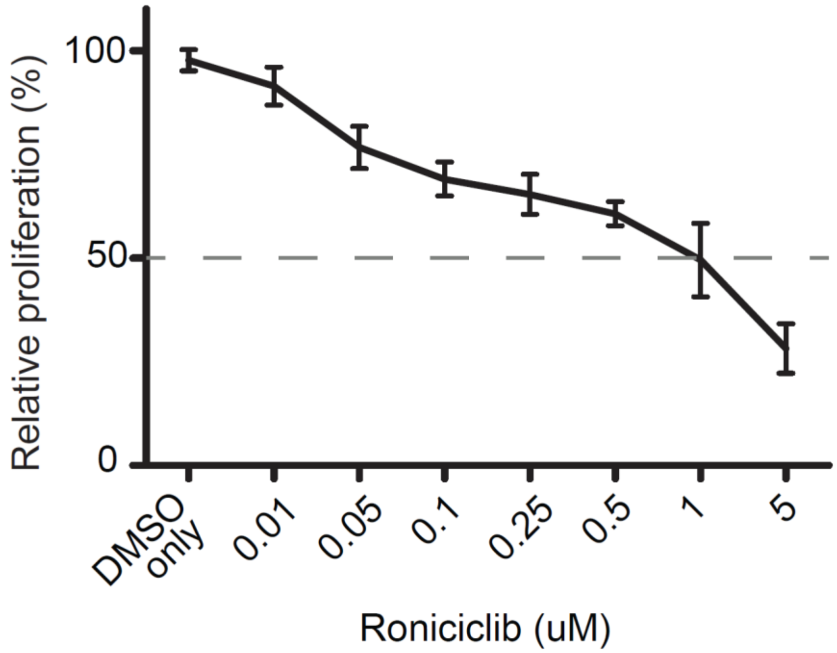

Figure S4. Roniciclib inhibits SY5Y cell proliferation. The SY5Y cells were exposed by different concentration of Roniciclib, and cell viability was measured at 72 h. The data represent the mean±SD of three independent experiments.

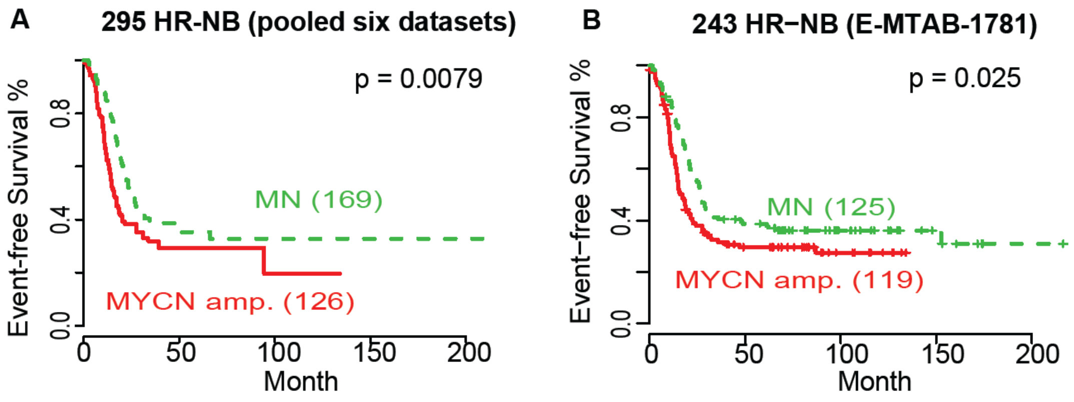

Figure S5. MYCN amplification has limited prognostic power for HR-NB patients.

A) Kaplan–Meier plots for the event-free survival of patients with HR-NB in the training dataset.

B) Kaplan–Meier plots for the event-free survival of patients with HR-NB in the validation dataset.

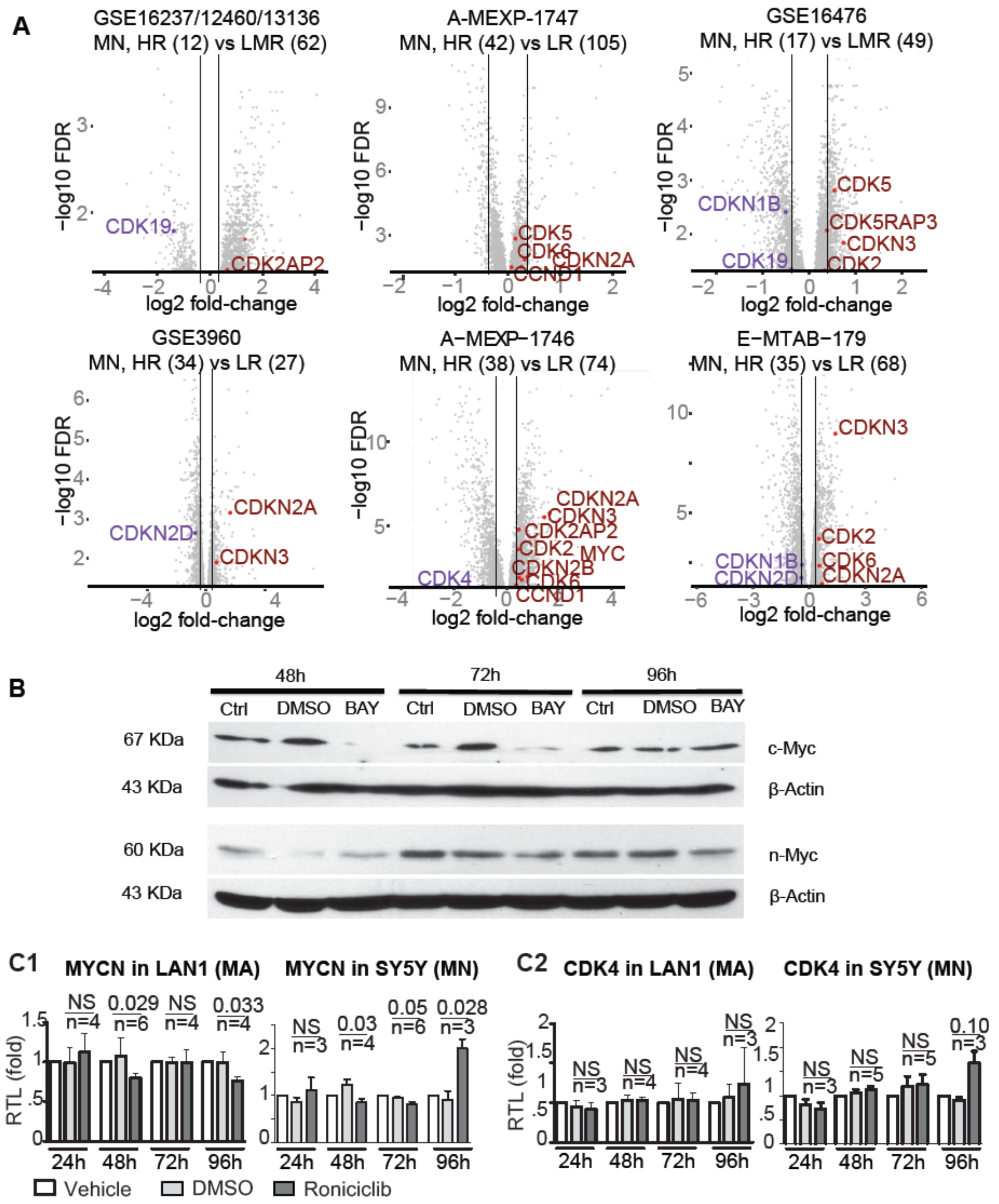

Figure S6. **The changes of gene expression in HR-NB cell lines and patients**.

A) Volcano-plots of significant gene expression changes in HR-NB compared with LR-NB. Each sub-panel represents one independent cohort, and the number of patients is given in parentheses. We used the limma test to identify differential expression from the expression profiles of only MN patients. When significant, we highlighted the expression change in genes CDKs, CDKNs, and CCND1.

B) Results of Western blotting for c-Myc and n-Myc. The LAN1 cells were stimulated by vehicle control (DMSO), BAY1000394 (1 μM) or left alone for the times indicated. The n-Myc protein levels were

analysed by immunoblotting using anti-n-Myc antibody (ThermoFisher, PA5-17403). β-actin was used as a loading control.

C) RT-PCR experimental results for MYCN and CDK4 in two HR-NB cell lines. Quantitative changes in mRNA expression was calculated between dimethyl sulfoxide (DMSO) and Roniciclib-treated cells. These relative expression levels were normalized to the mean of GAPDH. The data shown are the mean±SD of replicate experiments after normalization to the non-treated control, followed by the number of biological replicates. Statistics were calculated by a two-tailed Welch's corrected t-test.

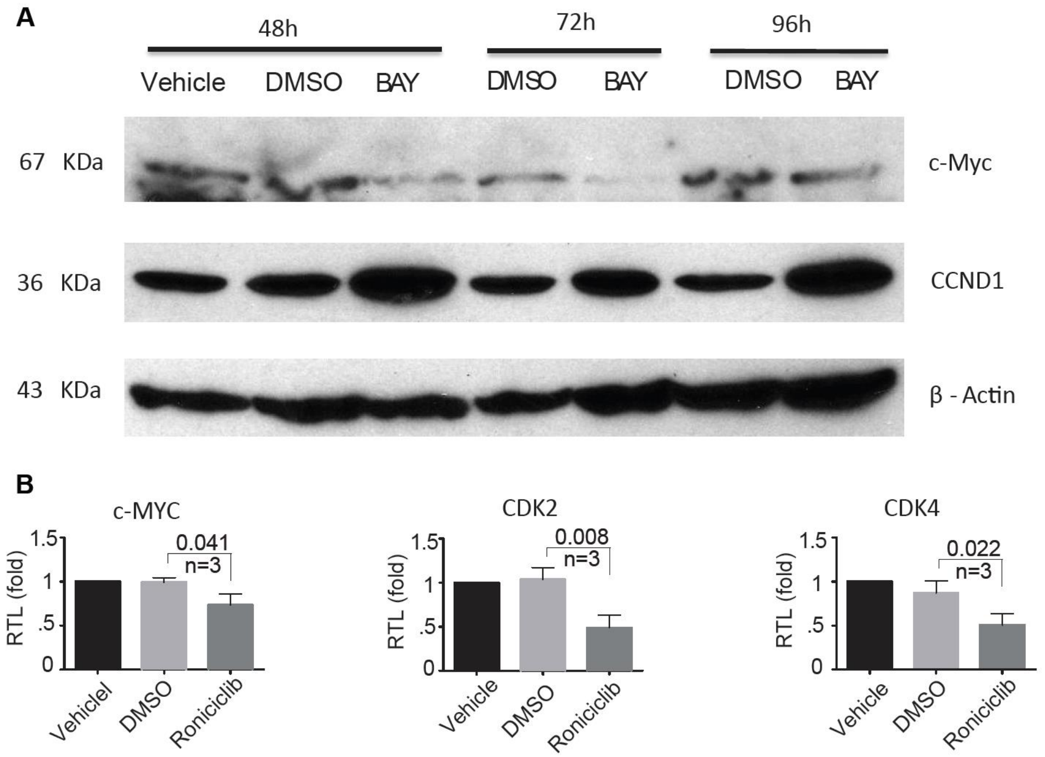

Figure S7. The effects of Roniciclib in the MCF7 breast cancer cells.

A) Results of Western blotting. The MCF7 cells were stimulated by vehicle control (DMSO), Roniciclib (1 μM) or left alone for 72 h. Expression levels of c-Myc and CCND1 proteins were analysed by immunoblotting using anti-c-Myc and anti-CCND1 antibodies. β-actin was used as an equal loading control.

B) Results of RT-PCR. The MCF7 cells were quantitatively analysed to examine mRNA levels between DMSO- and Roniciclib-treated cells. These relative expressions were normalized to the mean of GAPDH. The data present the mean±SD of replicate experiments after normalization to the non-treated control. The p-value of a two-tailed Welch's corrected t-test is given in each scenario, followed by the number of biological replicates.

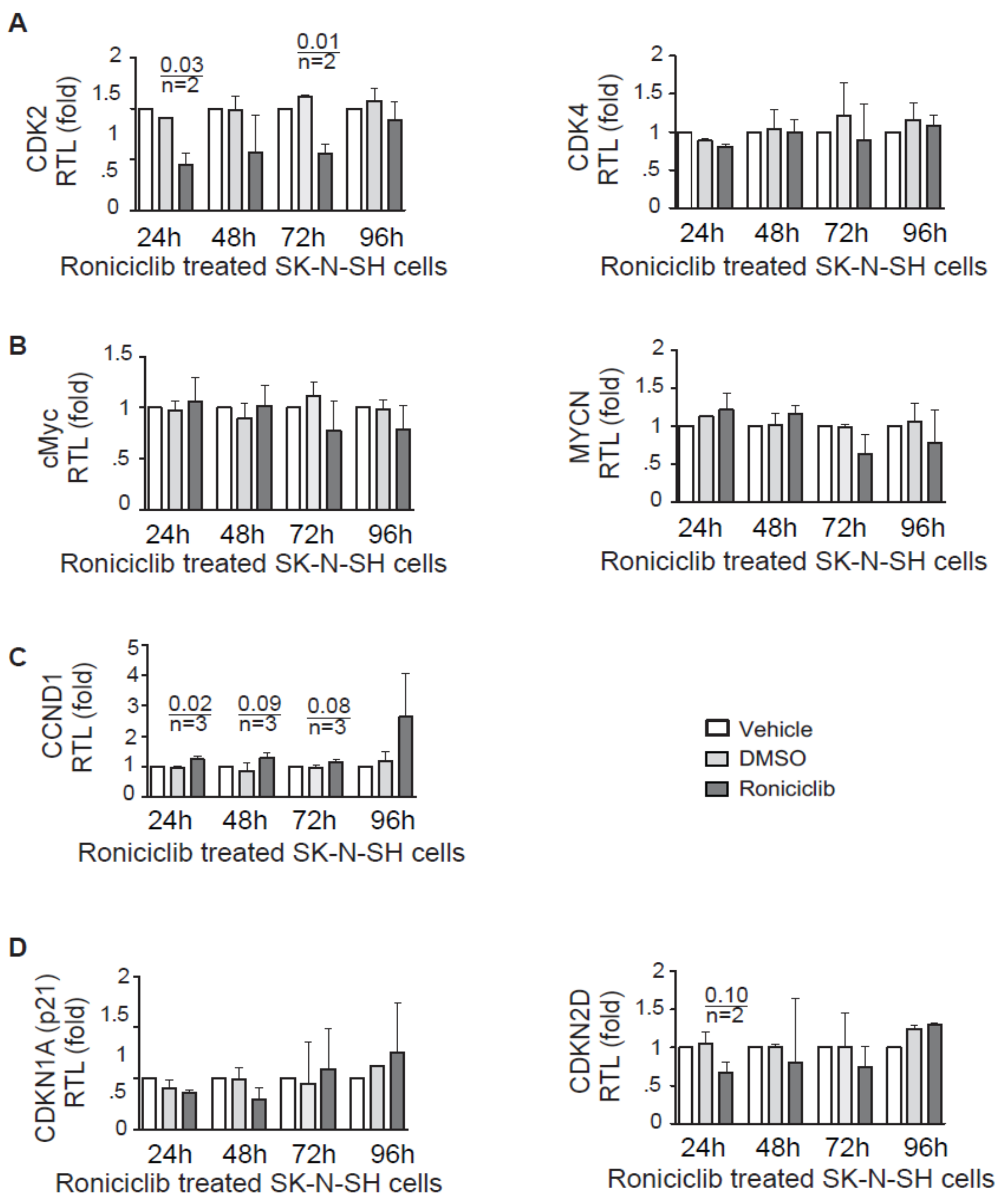

Figure S8. **The mRNA expression changes in SK-N-SH cells**.

A) Roniciclib selectively inhibits CDK2 rather than CDK4 in the SK-N-SH cells.

B) The mRNA expression of both c-Myc and n-Myc response to Roniciclib at 72 h in the SK-N-SH.

C) Roniciclib induces the mRNA expression of CCND1 significantly.

D) Roniciclib substantially decreases the mRNA expression of CDKN1A and CDKN2D along with CDK2 after an early phase (24 h) of treatment.

In each sub-panel, quantitative changes in mRNA expression was calculated between DMSO- and Roniciclib-treated cells. These relative expression levels were normalized to the mean of GAPDH. The data present the mean±SD of 2-3 replicate experiments after normalization to the non-treated control. Statistics were calculated by a two-tailed Student t-test, followed by the number of biological replicates.

Table S1. Summary of collected transcriptional data and clinical information for neuroblastoma.

Dataset	Derive MA and MN sig.	Derive HR sig.	prognostic index	HR size	HR MYCN-A (%)	Age (Month) *	INSS	MN pts size	HR MN size	LR MN size	FC cutoff (HR vs LR)	PMID	1st author	platform	Note for risk grouping/platform
**E-MTAB-161 / E-MTAB-179**	Y	Y	N	233	36 (15.4)	21.4±29	1,2,3,4,4s	112	38	74	1.3	20567016	Oberthuer	AMEXP-1746	There were 233 samples from which we excluded 87 pts with intermideate risk NB and 2 patients hard to classify its risk group.
**E-MTAB-161.1**	Y	N	Train	250	29 (11.6)	22.6±32		103	35	68	1.3	20676065	Oberthuer	AMEXP-1746	
**E-MTAB-161.2**	Y	Y	Train	271	35 (12.9)	23.8±35	1,2,3,4,4s	201	42	105	1.3	20567016	Oberthuer	AMEXP-1747	excluded 6 pts without risk clasification.
**GSE27608 / GSE21713**	Y	Y	Train	47	9 (19.1)	34.3±34	1,4	70	35*	25	1.3	21501490	Guo	exon	We categorized stage 4 samples with MN as "HMR" in this cohort to be compared with LR patients.
	Y	Y	Train	40	12 (30)	NA	1,2,3,4					21145484	Mestdagh		
**GSE3960**	Y	Y	Train	101	20 (19.8)	28.6±32	1,2,3,4	61	34	27	1.5	21124317	Wang	U95Av2	
**GSE16476**	Y	Y	Train	88	16 (18.2)	17.5±25	1,2,3,4,4s	31	17	49*	1.3	22367537	Molenaar	U133 plus2	There were 102 samples from which we excluded 20 pts with intermideate risk NB and 1 sample of brain tissues.
**GSE16237 / GSE13136 / GSE12460**	N	Y	Train	84				84	12	62*	1.8	20380745/17533364/18923523	Ohtaki/ Lastowska/ J-Lerosey	U133 plus2	The sampels were labbled as high, high-intermediate, intermediate, low-intermediate, and low group.
**E-MTAB-1781**	N	N	Validate	254	121 (17)	24.6±35	1,2,3,4,4s	580	128	367		25231397	Oberthuer	AMEXP-1746	562 pts had outcome following up
**E-TABM-38**	N	N	Validate	76	29 (38)	14.8±36	1,2,3,4,4s	218	47	131		17075126	Oberthuer	AMEXP-255	CDk19 was not measured
**# training pts (MA vs MN):**				**287**	**50**	**# training pts (HR vs LR):**			**178**	**299**					

#: we exclueded 8 overlaped, 1 withnot MYCN annotated, and 6 MYCN gain pts.

*: Pts with possiblly Intermediate-risk are considered due to lack of age and other clinical information.

For all other datasets, we excluded patients with intermideate risk and without MYCN status or the patients reported by the authors as "low-risk" but labelled with MYCN-amplification.

Table S2. MYC targets extracted from the Msigdb database.

MsigDB _ID	description; motif at the promoter regions [-2kb,2kb] around transcription start site	Pubmed	# of genes
**n-Myc potential targets in HR-NB or small cell lung cancer**			
LASTOWSKA_COAMPLIFIED_WITH_MYCN	Genes co-amplified within MYCN in primary neuroblastoma tumors	17533364	43
KIM_MYCN_AMPLIFICATION_TARGETS_UP	Genes positively correlated with amplifications of MYCN in small cell lung cancer cell lines.	16116477	92
KIM_MYCN_AMPLIFICATION_TARGETS_DN	Genes negatively correlated with amplifications of MYCN in small cell lung cancer cell lines.	16116477	103
WEI_MYCN_TARGETS_WITH_E_BOX	Genes whose promoters contain E-box motifs and whose expression changed in MYCN-3 cells (neuroblastoma) upon induction of MYCN	18504438	795
V$NMYC_01	NNCCACGTGNNN for MYCN		271
Sum of unique genes			**1259**
**c-Myc targets with motif match**			
V$MYCMAX_01	NNACCACGTGGTNN for MYC		255
V$MYCMAX_02	NANCACGTGNNW for MYC		268
V$MYCMAX_B	GCCAYGYGSN for MYC		268
V$MYCMAX_03	NNNNNNNCACGTGNNNNNNN for MYC		252
V$MYC_Q2	CACGTGS for MYC		185
CACGTG_V$MYC_Q2	CACGTG for MYC		1032
Sum of unique genes			**1275**
**MYC co-targe candidates**			
MYCN targets			1259
c_MYC targets with motif match			1277
a core set of 144 direct MYCN/c-MYC target genes in HR-NB (Westermann 2008)*	Additional data files 5-7		44
Sum of unique genes			**2257**

*: c-Myc/n-Myc targeting genes identified by ChIP-chip, microarray, and promoter motif analysis in NB cells [PMID: 18851746]

Table S3. The hub and bottleneck proteins, that were identified from PPI subnetworks pertaining to the HR signature but not the low-risk signature, highly involve the regulation of tumor-initiating cell (TIC) and cell cycle.

		PPI analysis				transcriptional analysis					functional gene-set		literature review	
Symbol	Category	recaptured by BioGRID	MA or MN subnetwork (STRING)	cMYC neighbor in the PPI	MYCN neighbor in the PPI	ESC-like cancer signature	MA signature	MN signature	HR signature	NB TIC over-express (Morozova et al)	cMYC.target	MYCN.target	PMID of selected ciataion	Summary
PLK1	TIC	Y	Y			Y	Y		Y	Y			21303981	PLK is a target for neuroblastoma tumor-initiating cells.
AURKA	TIC	Y	Y		Y	Y	Y		Y				24778030; 25268132; 23761169; 26782714	Targeting aurora kinase A inhibits hypoxia-mediated NB cell tumorigenesis; Aurora kinases as targets in drug-resistant neuroblastoma cells; AURKA enhanced cancer stemness phenotype in glioma and breast tumors.
CXCL12	TIC		Y					Y	Y				24124379	Activation of CXCL12/CXCR4 axis may be critical for different aspects of tumor initiation, progression, metastasis, and therapy resistance, and targeting CXCR4 signaling might be beneficial in cancer treatment.
RAF1	TIC	Y	Y	Y		Y	Y		Y				21215703	A clinical trial drug that inhibits RAF1-ERK signaling could prevent breast cancer progression by eliminating breast tumor initiating cells.
MYC (c-myc)	TIC		Y			Y		Y	Y				18851746; 24063893	High MYCN/c-MYC target gene expression is a hallmark of malignant NB progression. RNAi-mediated silencing of Myc transcription inhibits stem-like cell maintenance and tumorigenicity in prostate cancer.
STAT5A	TIC	Y	Y	Y				Y	Y				20161778; 16407271	Synergy between STAT5 and Gab2 was observed in HSC self-renewal, and the antiapoptotic effects of Epo in neuronal cells require the combinatorial activation of multiple signaling pathways, including STAT5. These results suggest that STAT5 might be exploited to optimize stem cell-based therapeutics.

CDK2	cell cycle		Y	Y			Y		Y		Y		19525400	Inactivation of CDK2 is synthetically lethal to MYCN over-expressing cancer cells.
CCNB1	cell cycle	Y	Y	Y			Y		Y				21159612	Combination of HDAC inhibitor and flavopiridol reduced CCNB1 in 2 cell lines (CHLA-90 and CHLA-172) with mt TP53 but not in those with wt TP53
CCND1	cell cycle	Y	Y	Y	Y	Y		Y	Y		Y		17384216	Cyclin D1, a novel molecular marker of minimal residual disease, in metastatic neuroblastoma
CDKN1A (p21)	cell cycle	Y	Y	Y				Y	Y				25958384	Forced expression of NB suppresor NBPF1 in two p53-mutant neuroblastoma cell lines resulted in a G1 cell cycle arrest and CDKN1A upregulation.
RAC2	cell cycle		Y	Y				Y	Y				25514883	Arrest cell cycle progression by inhibiting the activity of cyclin-dependent kinases. Regulation of transcription, apoptosis, DNA repair, as well as cell motility.
GRB2	cell cycle		Y					Y	Y			Y	18620523	A key molecule in intracellular signal transduction, linking activated cell surface receptors to downstream targets by binding to specific phosphotyrosine-containing and proline-rich sequence motifs.
BIRC5	cell cycle		Y	Y		Y	Y		Y	Y		Y	21859926	Knockdown of BIRC5 causes apoptosis in NB via mitotic catastrophe.
NRAS	cell cycle		Y	Y				Y	Y		Y	Y	23069660	Activated NRAS can stimulate a wide array of downstream effectors, mediating many cellular processes, such as proliferation, survival, invasion, endocytosis, cell-cell signaling and differentiation.
SMARCA4 (BRG1)	proliferation	Y	Y	Y			Y		Y			Y	26996667	Loss-of-function experiments in vitro and in vivo showed that SMARCA4 is essential for the proliferation of NB cells
PCNA	proliferation	Y	Y	Y		Y	Y		Y	Y			24728180	A PCNA-derived cell permeable peptide selectively inhibits NB cell growth.
CAD (CPS2)	proliferation		Y	Y			Y		Y		Y		12678497	To determine distinct cellular fates, including growth, differentiation and death.
GMPS	proliferation		Y			Y	Y		Y				24462112	GMPS is essential for cell proliferation and is required for stabilization of p53 and a TRIM21-GMPS-USP7 molecular cascade may regulate p53 levels.
POLR2B	cell cycle		Y				Y		Y				20855544	RPAP4/GPN1 is a POLR2B-associated protein that is essential for cell growth in eukaryotes and a member of a new family of GTPases.

PRKDC	NB marker	Y	Y	Y		Y	Y		Y				26716839	PRKDC encoded DNA-dependent protein kinase is molecular target for radiosensitization of NB cells.
EIF4E	NB marker		Y	Y			Y		Y		Y		15094766	EIF4E is overexpressed in several human cancers including NB makes it a prime target for anticancer therapies.
EIF4G1	NB marker		Y	Y			Y		Y		Y	Y	20669225	High expression of EIG4G1 was correlated with poor overall survival, and EIF4G1 hypermethylated exclusively in ganglioneuroblastoma than in NB.
NCBP2	NB marker		Y			Y	Y		Y				21124317	NCBP2 is potential downstream targets of LMO1, a neuroblastoma predisposition gene, and its expression level correlates with both LMO1 risk genotypes and copy number gains.
RPSA (LAMR1)	neuro-related	Y	Y			Y	Y		Y				16516231	A trans-dominant negative 37kDa/67kDa laminin receptor mutant impairs PrP(Sc) propagation in scrapie-infected neuronal cells.
POLR2E	cancer marker	Y	Y				Y		Y				25874495	Long noncoding RNAs POLR2E rs3787016 C/T and HULC rs7763881 A/C polymorphisms are associated with decreased risk of esophageal cancer.
LCK	cancer marker	Y	Y	Y				Y	Y				16186791	A member of the Src family of protein tyrosine kinases, plays a key role in T-lymphocyte activation and differentiation

PPI: protein-protein interaction; TIC: tunor initiating cell; MA: MYCN amplification; MN: MYCN normal copy; HR: high-risk.
